# Error Bounds for Dynamical Spectral Estimation

**DOI:** 10.1137/20m1335984

**Published:** 2021-02-11

**Authors:** Robert J. Webber, Erik H. Thiede, Douglas Dow, Aaron R. Dinner, Jonathan Weare

**Affiliations:** † Courant Institute of Mathematical Sciences, New York University, New York, NY 10012 USA.; ‡ Department of Chemistry, University of Chicago, Chicago, IL 60637 USA.; § Department of Mathematics, University of Chicago, Chicago, IL 60637 USA.

**Keywords:** transition operator, Rayleigh–Ritz method, Markov state models, computational statistical mechanics, conformation dynamics, 65C05, 60J35, 65N30

## Abstract

Dynamical spectral estimation is a well-established numerical approach for estimating eigenvalues and eigenfunctions of the Markov transition operator from trajectory data. Although the approach has been widely applied in biomolecular simulations, its error properties remain poorly understood. Here we analyze the error of a dynamical spectral estimation method called “the variational approach to conformational dynamics” (VAC). We bound the approximation error and estimation error for VAC estimates. Our analysis establishes VAC’s convergence properties and suggests new strategies for tuning VAC to improve accuracy.

## 1. Introduction.

An essential goal in simulation studies is to identify functions that decorrelate slowly in time. Since the values of these functions can be forecast far into the future, they are used for dimensionality reduction and prediction. Moreover, slowly decorrelating functions describe many scientifically significant processes. For example, in biomolecular systems, large-scale arrangements that control biological activity decorrelate slowly, compared to quickly fluctuating bond lengths and angles.

Dynamical spectral estimation is a numerical method that identifies slowly decorrelating functions by estimating the eigenfunctions and eigenvalues of the Markov transition operator of a system. Under appropriate assumptions, a small number of eigenfunctions span the most slowly decorrelating functions of the system, and the associated eigenvalues determine the slowest decorrelation rates. Dynamical spectral estimation uses simulated trajectories to estimate these quantities of interest.

Despite the wide acceptance of dynamical spectral estimation, estimated eigenfunctions and eigenvalues can have substantial error [45], and the cause of this error is not yet fully understood. Our goal here is to identify and bound the major error sources, thereby identifying opportunities where dynamical spectral estimation can produce accurate results.

Dynamical spectral estimation has been used in fields as diverse as biomolecular simulation [34], fluid mechanics [47], and geophysical analysis [9]. The approach goes by many names in the literature, including Markov state models [41], time-lagged independent component analysis [42], Ulam’s method [51], dynamical mode decomposition [40], and extended dynamical mode decomposition [54]. The methods are all closely related, so an error analysis for any one of the methods can shed useful light on the others. Here, for concreteness, we focus on a dynamical spectral estimation method that is well established in chemistry called “the variational approach to conformational dynamics” (VAC) [28, 5, 27, 13].

VAC can be applied to any Markov process *X*_*t*_ that is ergodic and reversible with respect to a distribution *μ*. Starting from a data set of simulated trajectories, VAC is applied in two steps. First, the data set is used to estimate expectations *C*_*ij*_(*τ*) = E_*μ*_ [*ϕ*_*i*_ (*X*_0_) *ϕ*_*j*_ (*X*_*τ*_)] involving a set of basis functions (*ϕ*_*i*_)_1≤*i*≤*n*_. Then, the spectral decomposition of the matrix *C* (0)^−1^ *C* (*τ*) is used to estimate eigenvalues and eigenfunctions of the transition operator of *X*_*t*_.

Our mathematical analysis establishes bounds on VAC’s approximation error and estimation error. *Approximation error* is the error in eigenvalue and eigenfunction estimates if the expectations *C*_*ij*_ (*τ*) = E_*μ*_ [*ϕ*_*i*_ (*X*_0_) *ϕ*_*j*_ *(X*_*τ*_)] are computed perfectly. *Estimation error* is the additional error incurred in VAC estimates because matrices *C* (0) and *C* (*τ*) are computed imperfectly using a finite data set.

We are not the first authors to mathematically examine VAC’s error. Djurdjevac and coauthors [7] bounded the approximation error of VAC eigenvalues. We extend their work by bounding the approximation error for VAC eigenfunctions, which are the chief objects of interest in most applications of dynamical spectral estimation. Additionally, we provide the first analysis of estimation error both for VAC eigenvalues and for eigenfunctions.

Our analysis of VAC also requires proving original error bounds. Standard bounds for the approximation of eigenspaces (e.g., [38, p. 103] or [18, p. 990]) depend on the inverse gap between eigenvalues. However, the gap between any two nontrivial eigenvalues of the transition operator vanishes exponentially fast with the lag time parameter *τ*. Therefore, the standard bounds increase exponentially as *τ* → ∞. In contrast to this asymptotic scaling, we contribute a sharp new perturbation bound that depends only on the inverse *relative* gap between eigenvalues. This new bound reaches its minimal value in the large *τ* limit, demonstrating the benefit of long lag times for reducing approximation error. In contrast, our asymptotic expressions for the estimation error do depend on the inverse spectral gap and grow in the large *τ* limit. Therefore, it is best to select an intermediate lag time.

While there is no single ideal lag time dictated by our analysis, we offer new tools for tuning VAC to reduce the estimation error. One such tool, the *VAC condition number*, identifies the subspaces of VAC eigenfunctions most sensitive to estimation error. A second diagnostic, the *mean squared estimation error*, identifies the typical size of the estimation error at different lag times. We provide data-driven formulas for calculating these quantities, enabling VAC users to identify and avoid the most problematic subspaces and lag times. Our experiments confirm that using these diagnostic tools leads to improved accuracy for VAC estimates.

The paper is organized as follows. Background material is given in section 2, theoretical results are in section 3, numerical experiments are in section 4, mathematical derivations are in section 5, and the conclusions follow in section 6.

## 2. Background.

This section presents background material explaining the VAC algorithm and the dynamical quantities VAC approximates.

### 2.1. VAC.

We begin by introducing the steps of VAC when the algorithm is applied to trajectory data from a Markov process *X*_*t*_ with an ergodic, reversible distribution *μ*. The algorithm starts by estimating expectations involving a set of basis functions (*ϕ*_*i*_)_1≤*i*≤*n*_. Subsequently, VAC solves an eigenvalue problem involving matrices of expectations.

**Algorithm 2.1:** VAC algorithm at lag time *τ*.

1. Form matrix $\hat{C}(0)$ with entries ${\hat{C}}_{ij}(0)\approx C_{ij}(0)={\text{E}}_{\mu }\left[\phi_{i}\left(X_{0}\right)\phi_{j}\left(X_{0}\right)\right]$.
2. Form matrix $\hat{C}(\tau )$ with entries ${\hat{C}}_{ij}(\tau )\approx C_{ij}(\tau )={\text{E}}_{\mu }\left[\phi_{i}\left(X_{0}\right)\phi_{j}\left(X_{\tau }\right)\right]$.
3. Solve eigenvalue problem ${\hat{\lambda }}_{i}^{\tau }{\hat{v}}^{i}(\tau )=\hat{C}{\left(0\right)}^{-1}\hat{C}(\tau ){\hat{v}}^{i}(\tau )$.
4. Return VAC eigenvalues ${\hat{\lambda }}_{i}^{\tau }$ and VAC eigenfunctions ${\hat{\gamma }}_{i}^{\tau }=\sum_{j}{\hat{v}}_{j}^{l}(\tau )\phi_{j}$.

In Algorithm 2.1, we are purposefully vague about the exact method for obtaining trajectory data to estimate

(2.1) $${\hat{C}}_{ij}(\tau )\approx C_{ij}(\tau )={\text{E}}_{\mu }\left[\phi_{i}\left(X_{0}\right)\phi_{j}\left(X_{\tau }\right)\right].$$

One common approach involves simulating long trajectories of *X*_*t*_ and removing the start of each trajectory to limit equilibration bias [43]. A second common approach (“importance sampling” [19]) involves simulating short trajectories and addressing bias through an appropriate reweighting procedure [31, 55]. Since there are no restrictions on how the data set is generated, enhanced sampling techniques can be used to generate the trajectory initial conditions or even the trajectories themselves [3, 32].

In addition to collecting a data set, another key design feature affecting VAC is the choice of basis functions. In the mid-1990s, early versions of VAC used the coordinate axes as basis functions [48, 11], a choice that remains common in molecular dynamics simulations [26, 42, 33]. Independently, in the late 1990s and early 2000s, researchers began constructing spectral estimates using “Markov state models” [41, 45, 46], a procedure mathematically equivalent to performing VAC using a basis of indicator functions on a partition of state space. This idea of using a basis of indicator functions can be traced back to a publication by Stanislaw Ulam in 1960 [51, pp. 74–75] and leads to simplifications in the eigenvalue problem in Algorithm 2.1. In the 2010s, it was observed that these schemes shared a common mathematical framework that could be extended to arbitrary basis sets [28]. Subsequent work led to the development of new families of basis functions [29, 53, 2, 30].

The name “variational approach to conformational dynamics” is inspired by the min-max principle for self-adjoint operators [28, 36]. This variational principle demonstrates that the top eigenfunctions *η*_1_, … , *η*_*k*_ of the transition operator maximize the value of the autocorrelation function

(2.2) $$\rho_{\eta }(\tau )={\text{corr}}_{\mu }\left[\eta \left(X_{0}\right),\eta \left(X_{\tau }\right)\right]$$

at any lag time *τ* ≥ 0. Thus, when *η* is a linear combination of the top *k* eigenfunctions and *u* is uncorrelated with the top *k* eigenfunctions, the autocorrelation functions are related by

(2.3) $$\rho_{\eta }(\tau )\geq \rho_{u}(\tau ),\;\tau \geq 0.$$

Consistent with this variational principle, VAC constructs linear combinations of basis functions that maximize autocorrelations. A recent approach due to [21] and [4] extends the linear fitting procedure in VAC by using artificial neural networks to maximize autocorrelations. However, in the present analysis we focus on the linear VAC algorithm as described in Algorithm 2.1, and we leave analysis of the nonlinear fitting procedure to future work.

To help clarify the relationship between VAC and other algorithms, we observe that the computational steps in Algorithm 2.1 can be used for many purposes. For example, AMUSE [50, 25] uses the same computational procedure as Algorithm 2.1, but the goal is to solve the blind-source separation problem in signal processing. Likewise, dynamic mode decomposition [37] and extended dynamic mode decomposition [54] use the same computational procedure as Algorithm 2.1, but the goal is to analyze nonreversible processes, particularly deterministic fluid flows. While the underlying computations are similar in all these cases, VAC refers specifically to the spectral estimation of time-reversible processes. To learn more about the connections between VAC and other related algorithms, we refer the reader to the helpful review paper by Klus and coauthors [16].

### 2.2. Spectral theory.

In this subsection, we take a closer look at the transition operator of the process *X*_*t*_ and its eigenfunctions. We assume *X*_*t*_ is either a continuous-time Feller process [14] or a discrete-time process restricted to even times *t* = 0, 2, 4,…. We assume *X*_*t*_ is ergodic and time-reversible with respect to a distribution *μ*. We use 〈·,·〉 to denote the inner product on the Hilbert space *L*^*2*^ (*μ*), and we set ∥·∥ = 〈·,·〉^1/2^. Lastly, we use $P_{U}$ to denote the orthogonal projection [35, p. 187] onto the closed linear subspace U and *P*_*f*_ to denote the orthogonal projection onto the one-dimensional subspace spanned by the function *f*.

The transition operator [14], also called the Koopman operator, is defined as the conditional expectation operator satisfying

(2.4) $$T_{t}[f](x)=\text{E}\left[f\left(X_{t}\right)|X_{0}=x\right].$$

There are three main properties of the transition operator that determine information about its eigenfunctions.

1. The transition operator *T*_*t*_ is self-adjoint in *L*^*2*^ (*μ*). The self-adjointness follows from the time-reversibility condition
  (2.5) $$\mu (dx)p_{t}(x,dy)=\mu (dy)p_{t}(y,dx),$$
  where *p*_*t*_ (*x*, *dy*) denotes the transition probabilities for the process *X*_*t*_. By integrating over (2.5), we verify the self-adjointness property
  (2.6) $$\langle f,T_{t}g\rangle =\langle T_{t}f,g\rangle ,\;f,g\in L^{2}(\mu ).$$
2. The transition operator satisfies the semigroup property
  (2.7) $$T_{t+s}\text{=}T_{t}\;T_{s}.$$
  For discrete-time processes, the semigroup property guarantees a decomposition
  (2.8) $$T_{t}={\left(T_{1}\right)}^{t},\;t=0,1,2,\ldots .$$
  For continuous-time Feller processes, the decomposition can be extended even further, leading to the formula
  (2.9) $$T_{t}=e^{tA},\;t\geq 0,$$
  which relates the semigroup *T*_*t*_ to its infinitesimal generator *A* [14].
3. The transition operator *T*_*t*_ is nonnegative, that is,
  (2.10) $$\langle f,T_{t}f\rangle =\langle T_{t/2}f,T_{t/2}f\rangle \geq 0,\;f\in L^{2}(\mu ),$$
  for all *t* ≥ 0 if *X*_*t*_ is a continuous-time process and for *t* = 0, 2, 4, … if *X*_*t*_ is a discrete-time process.

Using the spectral theorem for self-adjoint operators [35], we obtain a decomposition of either *A* or *T*_*2*_. Then, we extend this decomposition to the transition operator at all lag times *t* ≥ 0 or *t* = 0, 2, 4, …. The spectral decomposition takes the form

(2.11) $$T_{t}=\int_{0}^{\infty }e^{-\sigma t}\Pi (d\sigma ),$$

where ∏ (*dσ*) is a projection-valued measure.

The spectral decomposition completely determines the time correlations of the process *X*_*t*_. If the spectrum is discrete, then a finite set of orthonormal eigenfunctions is responsible for all the slowest decorrelations of the process. However, if there is an essential spectrum containing *σ* = 0, then an infinite set of orthonormal functions decorrelates arbitrarily slowly [35, p. 236].

**Figure 1.**
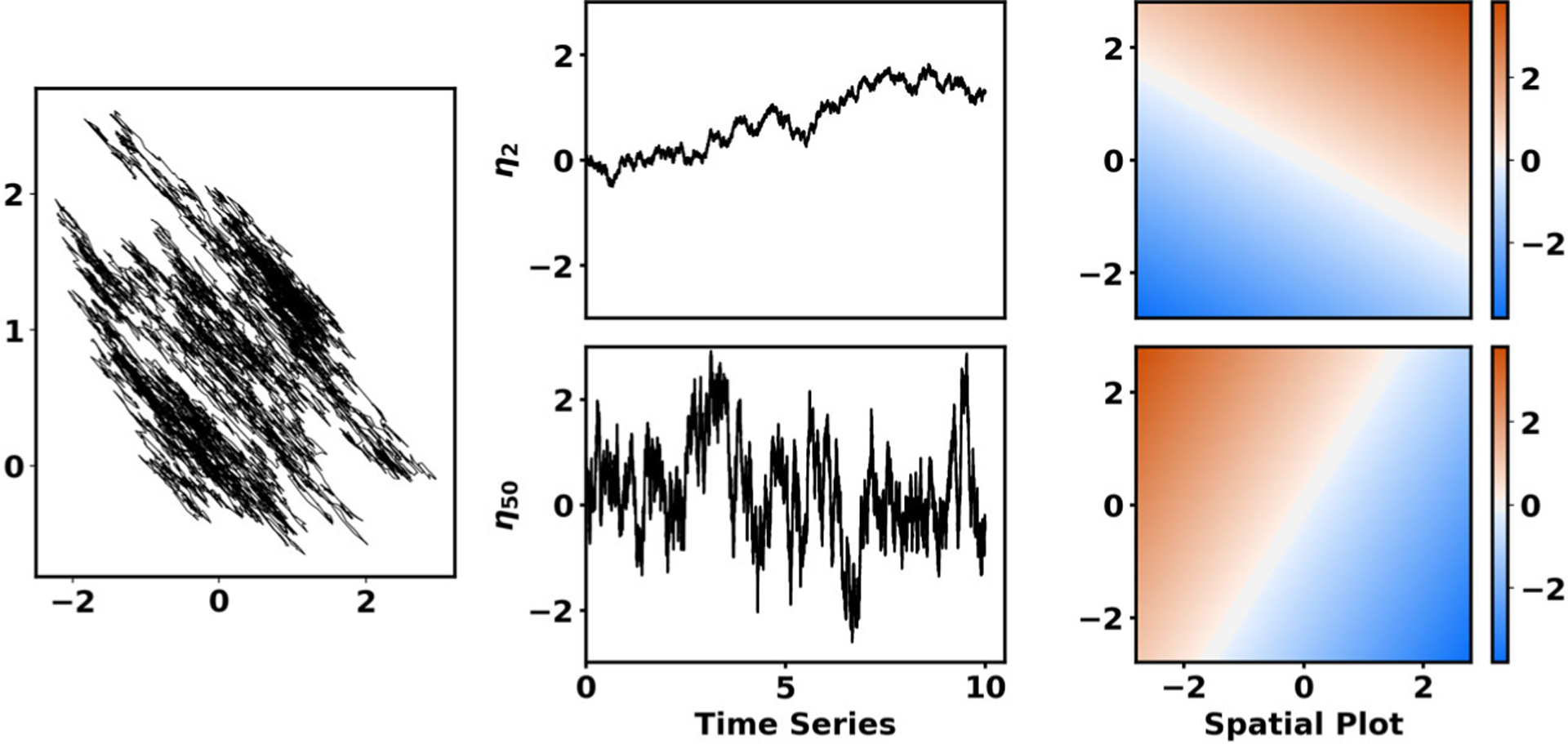
Eigenfunctions of a compact transition operator, corresponding to dynamics $d\left(\begin{array}{l} X_{t}\\ Y_{t} \end{array}\right)=\left(\begin{array}{cc} -0.4 & 0.17\\ 0.17 & -0.2 \end{array}\right)\left(\begin{array}{c} X_{t}\\ Y_{t} \end{array}\right)dt+\sqrt{2}d\left(\begin{array}{c} W_{t}^{1}\\ W_{t}^{2} \end{array}\right)$. Left: Typical trajectory of (X_t_, Y_t_). Upper middle: Time series for eigenfunction η_2_ with slow decorrelation timescale $\sigma_{2}^{-1}=5$. Lower middle: Time series for eigenfunction η_50_ with fast decorrelation timescale $\sigma_{50}^{-1}=0.1$. Right: Spatial structure of η_2_ and η_50._

To avoid the possibility of having an essential spectrum containing *σ* = 0, it is sufficient to assume that *T*_*t*_ is compact. Under compactness, the spectral decomposition takes the form

(2.12) $$T_{t}=\sum_{i=1}^{\infty }e^{-\sigma_{i}t}P_{\eta_{i}},$$

where $e^{-\sigma_{1}t}>e^{-\sigma_{2}t}\geq e^{-\sigma_{3}t}\geq \cdots$ are eigenvalues and *η*_1_, *η*_2_, *η*_3_, … are the associated eigenfunctions. Since the process is ergodic, $e^{-\sigma_{1}t}=1$ is a simple eigenvalue of *T*_*t*_ corresponding to the eigenfunction *η*_1_ = 1. Figure 1 shows additional examples of eigenfunctions for a compact transition operator *T*_*t*_.

While the compactness assumption is enough to facilitate a rigorous analysis of VAC, the compactness assumption can be overly restrictive. In the Monte Carlo literature, there are numerous examples of transition operators that are not compact, such as the transition operator for the Metropolis–Hastings sampler [23, 1]. Therefore, we prefer to use the quasi-compactness assumption, a weaker assumption satisfied by a broader class of processes.

*Assumption* 2.1 (quasi-compactness). The spectral decomposition for the transition operator *T*_*t*_ takes the form

(2.13) $$T_{t}=\sum_{i=1}^{r}e^{-\sigma_{i}t}P_{\eta_{i}}+R_{t},\;R_{t}=\int_{\sigma_{r+1}}^{\infty }e^{-\sigma t}\Pi (d\sigma ).$$

Here, $e^{-\sigma_{1}t}>e^{-\sigma_{2}t}\geq \cdots \geq e^{-\sigma_{r}t}$ are eigenvalues, *η*_1_, *η*_2_, … , *η*_*r*_ are the associated eigenfunctions, and $e^{-\sigma_{r+1}t}$ is not necessarily an eigenvalue but it bounds the operator norm of the residual operator, that is, ${\left\|R_{t}\right\|}_{2}\leq e^{-\sigma_{r+1}t}$.

*Remark* 2.2. In the analysis to follow, an “eigenspace” of *T*_*t*_ denotes the closed linear subspace of eigenfunctions with a given eigenvalue. An “invariant subspace” U is any closed linear subspace satisfying $T_{t}U\subseteq U$.

*Remark* 2.3. There is a common modification of Algorithm 2.1 where the estimated mean ${\hat{\mu }}_{i}\approx \mu_{i}={\text{E}}_{\mu }\left[\phi_{i}\left(X_{0}\right)\right]$ is subtracted from each one of the basis functions *ϕ*_*i*_ before performing VAC (see the discussion in [16]). When the mean is removed, VAC no longer estimates the trivial eigenfunction *η*_1_ = 1 with eigenvalue $e^{-\sigma_{1}t}=1$; however, VAC continues to estimate all other eigenvalues and eigenspaces.

### 2.3. Approximation of eigenspaces.

It is colloquially said that VAC approximates eigenvalues and eigenfunctions, but it is more correct to say that VAC approximates eigenvalues and *eigenspaces*. Recall that ${\hat{\lambda }}_{i}^{\tau }$ and ${\hat{\gamma }}_{i}^{\tau }$ are the VAC eigenvalues and eigenfunctions, while $e^{-\sigma_{i}\tau }$ and *η*_*i*_ the true eigenvalues and eigenfunctions of the transition operator. We assume that VAC eigenvalues are arranged from largest to smallest so that ${\hat{\lambda }}_{1}^{\tau }\geq {\hat{\lambda }}_{2}^{\tau }\geq \cdots \geq {\hat{\lambda }}_{n}^{\tau }$. Then VAC approximates eigenvalues

(2.14) $${\hat{\lambda }}_{i}^{\tau }\approx e^{-\sigma_{i}\tau },\;1\leq i\leq n.$$

VAC approximates eigenspaces and other invariant subspaces

(2.15) $$\underset{j\leq i\leq k}{\text{span}}{\hat{\gamma }}_{i}^{\tau }\approx \underset{j\leq i\leq k}{\text{span}}\eta_{i}$$

whenever there is a gap between {*σ*_*j*_, … , *σ*_*k*_} and all other *σ*_*i*_ values.

To measure the error in VAC’s invariant subspaces, we introduce two distances: the gap distance *d*_2_ (·, ·) and the projection distance *d*_F_ (·, ·) [8].

Definition 2.4. *Consider closed subspaces U and W, and let $W^{⊥}$ indicate the orthogonal complement of W. Then, we define the gap distance and projection distance as follows:*

(2.16) $$d_{2}(U,W)={\left\|P_{W^{⊥}}P_{U}\right\|}_{2},\;d_{\text{F}}(U,W)={\left\|P_{W^{⊥}}P_{U}\right\|}_{\text{F}}.$$

*Here*, ∥·∥_2_ *denotes the operator norm, and* ∥·∥_F_ *denotes the Hilbert*–*Schmidt norm, also known as the Frobenius norm*.

The gap distance and projection distance are very flexible, and definitions (2.16) can be applied even if dim(U)<dim(W)≤∞. In this case, we observe that $d_{2}(U,W)$ and $d_{\text{F}}(U,W)$ are not technically distances. Rather, $d_{2}(U,W)$ and $d_{\text{F}}(U,W)$ are properly interpreted as distances between U and the nearest (U)-dimensional  subspace of W.

We end this section by introducing a useful property of the projection distance, which we apply repeatedly in the analysis.

Lemma 2.5. *Consider* $U=\text{span}\left(U_{1},U_{2}\right)$, *where* $U_{1}$ *and* $U_{2}$ *are orthogonal subspaces, and* $W=\text{span}\left(W_{1},W_{2}\right)$, *where* $W_{1}$ *and* $W_{2}$ *are orthogonal subspaces. Then*,

(2.17) $$d_{\text{F}}^{2}\left(U_{2},W_{2}\right)\leq d_{\text{F}}^{2}(U,W)+d_{\text{F}}^{2}\left(U_{1},W_{1}\right).$$

*Proof*. Calculate

$$\begin{array}{lll} \left(2.18\right) & d_{\text{F}}^{2}\left(U_{2},W_{2}\right) & ={\left\|P_{U_{2}}P_{W^{⊥}}\right\|}_{\text{F}}^{2}+{\left\|P_{U_{2}}P_{W_{1}}\right\|}_{\text{F}}^{2}\\ \left(2.19\right) & & \leq {\left\|P_{U}P_{W^{⊥}}\right\|}_{\text{F}}^{2}+{\left\|P_{U_{1}^{⊥}}P_{W_{1}}\right\|}_{\text{F}}^{2}\\ \left(2.20\right) & & =d_{\text{F}}^{2}(U,W)+d_{\text{F}}^{2}\left(U_{1},W_{1}\right). \end{array}$$

■

## 3. Theoretical results.

To describe the approach taken in the theoretical analysis, we introduce an idealized VAC algorithm where expectations *C*_*ij*_ (*τ*) = E_*μ*_ [*ϕ*_*i*_ (*X*_0_) *ϕ*_*j*_ (*X*_*τ*_)] and *C*_*ij*_ (0) = E_*μ*_ [*ϕ*_*i*_ (*X*_0_) *ϕ*_*j*_ (*X*_0_)] are computed perfectly. Notationally, we distinguish between VAC and idealized VAC by using the ^ symbol to indicate the quantities calculated using data. For VAC, we write ${\hat{C}}_{ij}(\tau )$, ${\hat{v}}^{i}(\tau )$, ${\hat{\lambda }}_{i}^{\tau }$, and ${\hat{\gamma }}_{i}^{\tau }$. For idealized VAC, we write *C*_*ij*_ (*τ*), *υ*^*i*^ (*τ*), $v^{i}(\tau )$, and $\lambda_{i}^{\tau }$.

In the theoretical analysis, we use idealized VAC to isolate two different sources of error. We decompose the subspace error using

(3.1) $$\underset{\text{total error }}{\underset{︸}{d_{\text{F}}\left(\underset{j\leq i\leq k}{\text{span}}{\hat{\gamma }}_{i}^{\tau },\underset{j\leq i\leq k}{\text{span}}\eta_{i}\right)}}\leq \underset{\text{approximation error }}{\underset{︸}{d_{\text{F}}\left(\underset{j\leq i\leq k}{\text{span}}\gamma_{i}^{\tau },\underset{j\leq i\leq k}{\text{span}}\eta_{i}\right)}}+\underset{\text{estimation error }}{\underset{︸}{d_{\text{F}}\left(\underset{j\leq i\leq k}{\text{span}}{\hat{\gamma }}_{i}^{\tau },\underset{j\leq i\leq k}{\text{span}}\gamma_{i}\right)}}.$$

Analogously, we decompose the eigenvalue error using

(3.2) $$\underset{\text{total error }}{\underset{︸}{\left|{\hat{\lambda }}_{i}^{\tau }-e^{-\sigma_{i}\tau }\right|}}\leq \underset{\text{approximation error }}{\underset{︸}{\left|\lambda_{i}^{\tau }-e^{-\sigma_{i}\tau }\right|}}+\underset{\text{estimation error }}{\underset{︸}{\left|{\hat{\lambda }}_{i}^{\tau }-\lambda_{i}^{\tau }\right|}}.$$

Approximation error is the difference between idealized VAC estimates and the true eigenvalues and eigenspaces. Estimation error is the difference between VAC estimates and idealized VAC estimates. We first present approximation error bounds in subsection 3.1, and then we present estimation error bounds in subsection 3.2.

**Figure 2.**
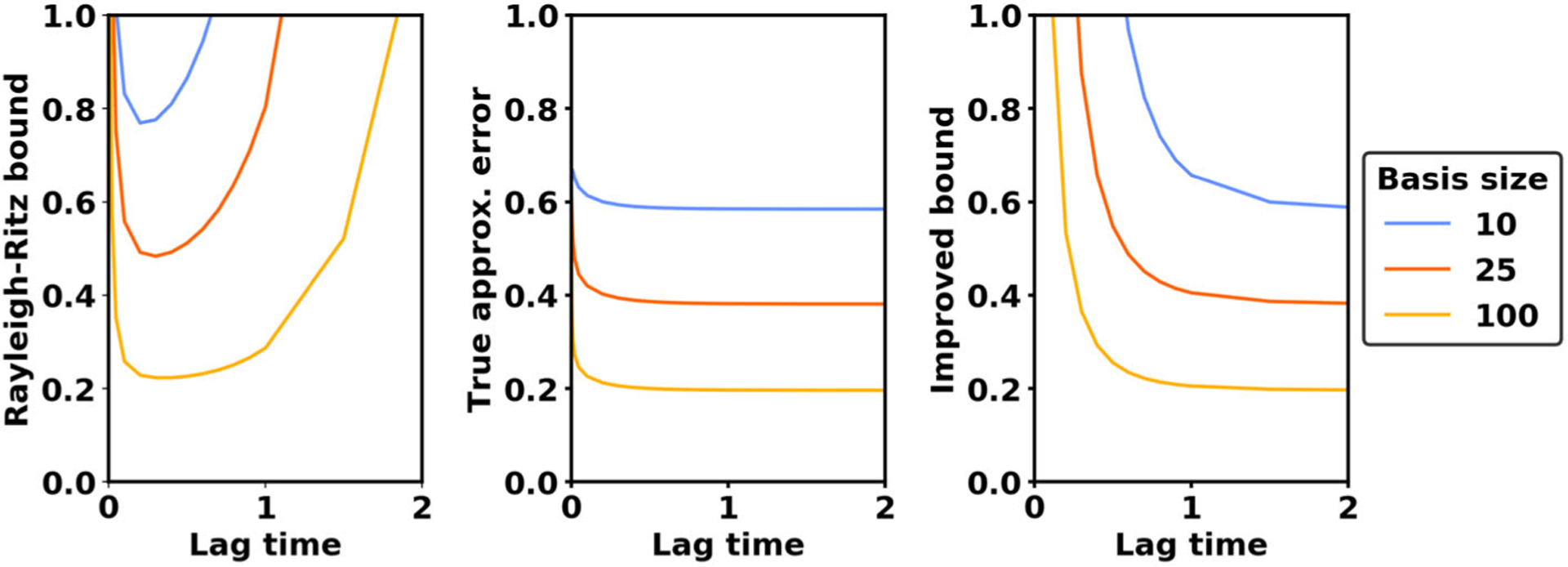
Comparison between different bounds for the approximation error. Left: The Rayleigh–Ritz bound asymptotes to infinity at long and short lag times. Center: The true approximation error stabilizes at long lag times. Right: The improved bound presented in Theorem 3.4 is asymptotically sharp at long lag times.

**Figure 3.**
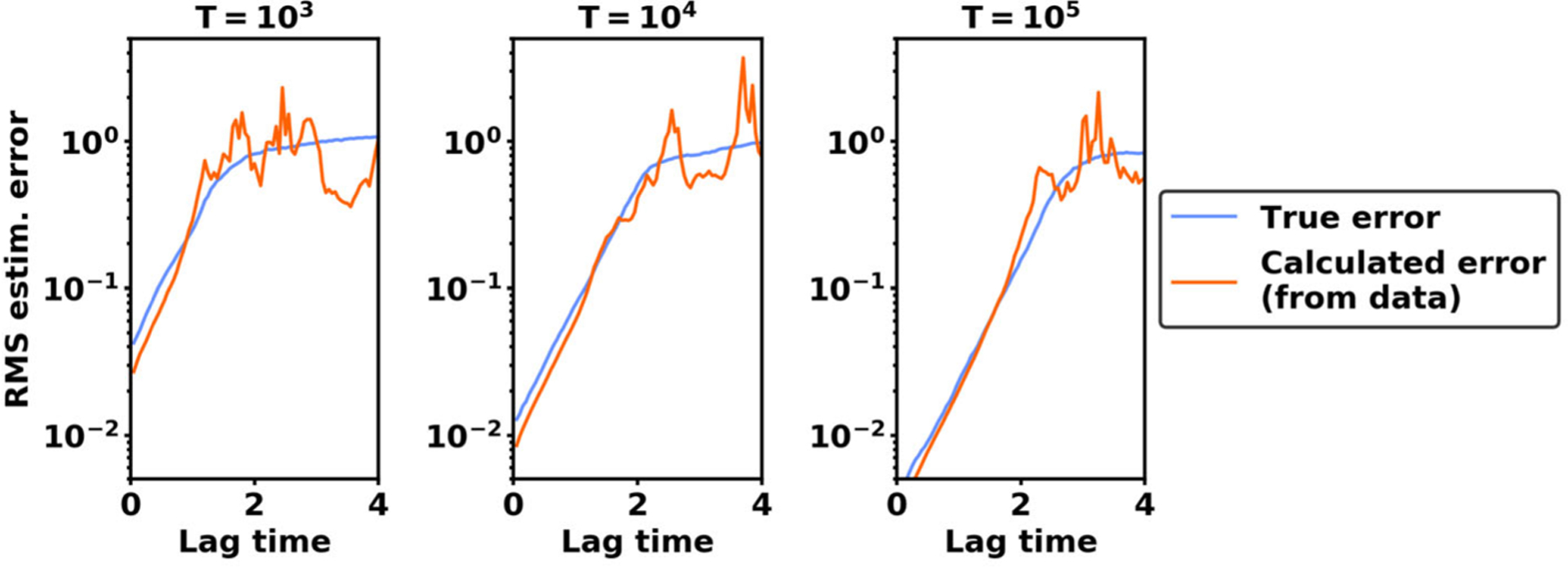
Root mean squared estimation error for different trajectory lengths T. The calculated error is obtained from a single trajectory of data using formulas in Theorem 3.7. The true error is obtained through 100 independent trials.

*Remark* 3.1. To illustrate the implications of our error bounds, we use numerical experiments. Thus, Figure 2 and Figure 3 demonstrate the error of VAC when applied to the Ornstein–Uhlenbeck (OU) process $dX=-X\;dt+\sqrt{2}\;dW$ using a basis of indicator functions. Details on how the figures were generated appear in the accompanying supplemental material file SMM133598.pdf [local/web 301KB].

### 3.1. Approximation error.

In this subsection, we first bound the approximation error by using traditional Rayleigh–Ritz approximation bounds. However, we find that the Rayleigh–Ritz bounds do not provide enough information to show how approximation error depends on the lag time parameter *τ*. Therefore, we derive improved bounds by using original methods. The improved bounds are asymptotically sharp at long lag times, revealing that long lag times cause the approximation error to stabilize.

#### 3.1.1. Existing approximation bounds are inadequate.

The idealized VAC algorithm is equivalent to the *Rayleigh-Ritz method* in spectral estimation. In the Rayleigh–Ritz method [44], the eigenvalues and eigenspaces of a target operator *A* are estimated by introducing a subspace of functions U and then calculating the eigenvalues and eigenspaces of ${P_{U}A|}_{U}$, where ${A|}_{U}$ denotes the restriction of *A* to the subspace U. This is also exactly what is done in idealized VAC. The target operator is the transition operator *T*_*τ*_, and the subspace of basis functions is Φ = span_1≤*i*≤*n*_ *ϕ*_*i*_. Moreover, the idealized VAC eigenfunctions $\gamma_{i}^{\tau }$ are eigenfunctions of *P*_Φ_ *T*_*τ*_|_Φ_ with eigenvalues $\lambda_{i}^{\tau }$.

The equivalence between the Rayleigh–Ritz method and idealized VAC is known in the VAC literature [39, 7]. However, the implications for VAC’s approximation error have not yet been fully explored. Djurdjevac and coauthors [7] applied Rayleigh–Ritz error bounds to analyze idealized VAC eigenvalues. The following theorem goes a step further, by also applying Rayleigh–Ritz error bounds to analyze idealized VAC eigenspaces.

Theorem 3.2 (approximation bounds). *Fix the lag time τ* > 0 *and the index* 1 ≤ *k* ≤ *r*, *but allow the basis set* Φ *to vary. In the limit as d*_F_ (span_1≤*i*≤*k*_ *η*_*i*_, Φ) → 0, *the idealized VAC estimates converge as follows:*

1. *The idealized VAC eigenvalues* 1, 2, … , *k all converge as*
  (3.3) $$\lambda_{i}^{\tau }\to e^{-\sigma_{i}\tau },\;1\leq i\leq k.$$
2. *When there is a gap between* {*σ*_*j*_, … , σ_*k*_} *and all other σ*_*i*_ *values, the subspace* ${\text{span}}_{j\leq i\leq k}\gamma_{i}^{\tau }$ *of idealized VAC eigenfunctions converges as*
  (3.4) $$\underset{j\leq i\leq k}{\text{span}}\gamma_{i}^{\tau }\to \underset{j\leq i\leq k}{\text{span}}\eta_{i}.$$
  *Additionally, error bounds are given as follows:*
  1. *The kth idealized VAC eigenvalue is bounded by*
    (3.5) $$1-d_{2}^{2}\left(\underset{1\leq i\leq k}{\text{span}}\eta_{i},\Phi \right)\leq \frac{\lambda_{k}^{\tau }}{e^{-\sigma_{k}\tau }}\leq 1.$$
  2. *The top k idealized VAC eigenfunctions are bounded by*
    (3.6) $$1\leq \frac{d_{\text{F}}^{2}\left(\underset{1\leq i\leq k}{\text{span}}\gamma_{i}^{\tau },\underset{1\leq i\leq k}{\text{span}}\eta_{i}\right)}{d_{\text{F}}^{2}\left(\underset{1\leq i\leq k}{\text{span}}\eta_{i},\Phi \right)}\leq 1+\frac{{\left\|P_{\Phi^{⊥}}T_{\tau }P_{\Phi }\right\|}_{2}^{2}}{{\left|e^{-\sigma_{k}\tau }-\lambda_{k+1}^{\tau }\right|}^{2}}.$$

*Proof*. See [17, 18] for the original proofs, or see the derivations in the accompanying supplemental material file SMM133598.pdf [local/web 301KB]. ■

The main takeaway from Theorem 3.2 is that the approximation error converges to zero in the limit as

(3.7) $$d_{\text{F}}\left(\underset{1\leq i\leq k}{\text{span}}\eta_{i},\Phi \right)\to 0.$$

Condition (3.7) implies that the basis set Φ must become very rich, so that eigenfunctions *η*_*i*_ can be closely approximated using linear combinations of basis functions.

The Rayleigh–Ritz error bound (3.6) clearly indicates that the eigenspace approximation error must decay with an increasingly rich basis. However, the bound is not sufficiently detailed to show how the approximation error depends on the lag time *τ*. As seen in Figure 2, the Rayleigh–Ritz bound (3.6) asymptotes to infinity as the lag time increases, implying that approximation error can grow arbitrarily large. In contrast to this upper bound, however, experiments reveal that the approximation error decreases and then stabilizes as the lag time tends to infinity. In the next section, we will derive an improved bound that is asymptotically sharp, describing the exact behavior of the approximation error as *τ* → ∞.

#### 3.1.2. New approximation bounds.

To analyze the dependence on lag time, we develop a mathematical approach different from the methods applied to the Rayleigh–Ritz method in the past. We start by identifying a key stability property of idealized VAC that has not appeared in the previous literature. As *τ* → ∞, idealized VAC eigenspaces converge to a well-defined limit, implying that the approximation error must stabilize at long lag times.

To rigorously study the convergence of idealized VAC estimates, our first step is to introduce the orthogonalized projection functions *q*_1_, *q*_2_, …. These are the natural functions to appear in the *τ* → ∞ limit. They are constructed from the projected eigenfunctions *P*_Φ_*η*_1_, *P*_Φ_*η*_2_, … , but they are adjusted to meet the orthogonality constraints on idealized VAC eigenfunctions.

Definition 3.3. *Set p* = min {*n*, *r*}, *where n is the number of basis functions* (*ϕ*_*i*_)_1≤*i*≤*n*_ *and r is the number of eigenfunctions* (*η*_*i*_)_1≤*i*≤*r*_ *identified in* Assumption 2.1. *Assume that projections P*_Φ_*η*_*i*_ *are linearly independent for* 1≤ *i ≤ p*. *Then, define*

$$\begin{array}{lll} \left(3.8\right) & {\tilde{q}}_{1}=P_{\Phi }\eta_{1}, & q_{1}={\tilde{q}}_{1}/\left\|{\tilde{q}}_{1}\right\|,\\ \left(3.9\right) & {\tilde{q}}_{2}=P_{\Phi }\eta_{2}-\langle q_{1},\eta_{2}\rangle q_{1}, & q_{2}={\tilde{q}}_{2}/\left\|{\tilde{q}}_{2}\right\|,\\ \left(3.10\right) & \vdots & \\ \left(3.11\right) & {\tilde{q}}_{p}=P_{\Phi }\eta_{p}-\sum_{i=1}^{p-1}\langle q_{i},\eta_{p}\rangle q_{i}, & q_{p}={\tilde{q}}_{p}/\left\|{\tilde{q}}_{p}\right\|. \end{array}$$

Our next step is to prove that idealized VAC eigenfunctions $\gamma_{i}^{\tau }$ converge to the orthogonalized projections *q*_*i*_ at long lag times.

Theorem 3.4 (the *τ* → ∞ limit). *Fix the basis set* Φ *but allow the lag time τ to vary. In the limit as τ* → ∞, *idealized VAC estimates converge as follows:*

1. *When there is a gap between σ*_*k*_ *and all other σ*_*i*_ *values, the kth idealized VAC eigenvalue satisfies*
  (3.12) $$\frac{\lambda_{k}^{\tau }}{e^{-\sigma_{k}\tau }}\to {\langle \eta_{k},q_{k}\rangle }^{2}.$$
2. *When there is a gap between* {*σ*_*j*_, … , *σ*_*k*_} *and all other σ*_*i*_ *values, the subspace* ${\text{span}}_{j\leq i\leq k}\gamma_{i}^{\tau }$ *of idealized VAC eigenfunctions converges as*
  (3.13) $$\underset{j\leq i\leq k}{\text{span}}\gamma_{i}^{\tau }\to \underset{j\leq i\leq k}{\text{span}}q_{i}.$$
3. *When there is a gap between σ*_*k*_ *and all other σ*_*i*_ *values and a gap between σ*_k+1_ *and all other σ*_*i*_ *values, the top k idealized VAC eigenfunctions satisfy*
  (3.14) $$d_{\text{F}}\left(\underset{1\leq i\leq k}{\text{span}}\gamma_{i}^{\tau },\underset{1\leq i\leq k}{\text{span}}q_{i}\right)\frac{\lambda_{k}^{\tau }}{\lambda_{k+1}^{\tau }}\to \left|\frac{\langle \eta_{k+1},q_{k}\rangle }{\langle \eta_{k+1},q_{k+1}\rangle }\right|.$$

*Proof*. See subsection 5.2, subsection 5.3, and subsection 5.4. ■

The main message of Theorem 3.4 is that idealized VAC eigenspaces converge exponentially fast as *τ* → ∞. Because of this convergence, the approximation error must stabilize. As the last step of our approximation error analysis, we use the stabilization at long lag times to provide a new, asymptotically sharp bound on VAC’s approximation error.

Theorem 3.5. *When* $\lambda_{k}^{\tau }>e^{-\sigma_{k+1}\tau }$, *the top k idealized VAC eigenfunctions are bounded by*

(3.15) $$1\leq \frac{d_{\text{F}}^{2}\left(\underset{1\leq i\leq k}{\text{span}}\gamma_{i}^{\tau },\underset{1\leq i\leq k}{\text{span}}\eta_{i}\right)}{d_{\text{F}}^{2}\left(\underset{1\leq i\leq k}{\text{span}}\eta_{i},\Phi \right)}\leq 1+\frac{1}{4}{\left|\frac{e^{-\sigma_{k+1}\tau }}{\lambda_{k}^{\tau }-e^{-\sigma_{k+1}\tau }}\right|}^{2}.$$

*Proof* See subsection 5.3. ■

Interpreting the results of this subsection, we can identify concrete strategies for how best to reduce approximation error. The approximation error can be divided into two parts:

(3.16) $$\underset{\text{approximation error }}{\underset{︸}{d_{\text{F}}\left(\underset{j\leq i\leq k}{\text{span}}\gamma_{i}^{\tau },\underset{j\leq i\leq k}{\text{span}}\eta_{i}\right)}}\leq \underset{\text{lag-time-independent error }}{\underset{︸}{d_{\text{F}}\left(\underset{j\leq i\leq k}{\text{span}}q_{i},\underset{j\leq i\leq k}{\text{span}}\eta_{i}\right)}}+\underset{\text{lag-time-dependent error }}{\underset{︸}{d_{\text{F}}\left(\underset{j\leq i\leq k}{\text{span}}\gamma_{i}^{\tau },\underset{j\leq i\leq k}{\text{span}}q_{i}\right)}}.$$

In this decomposition, we separate the lag-time-independent error and the lag-time-dependent error. In applications of VAC, there are separate strategies for reducing these two error sources.

To reduce the lag-time-independent error, the best strategy is to enrich the basis set as much as possible. If the basis set is rich enough to approximate the top eigenfunctions *η*_*1*_, *η*_2_, … , *η*_*k*_ with high accuracy, then the lag-time-independent error must be low. Assuming there is a gap between {*σ*_*j*_, … , *σ*_*k*_} and all other *σ*_*i*_ values, Lemma 2.5 guarantees

(3.17) $$\underset{\text{squared lag-time-independent error }}{\underset{︸}{d_{\text{F}}^{2}\left(\underset{j\leq i\leq k}{\text{span}}q_{i},\underset{j\leq i\leq k}{\text{span}}\eta_{i}\right)}}\leq d_{\text{F}}^{2}\left(\underset{1\leq i\leq j-1}{\text{span}}\eta_{i},\Phi \right)+d_{\text{F}}^{2}\left(\underset{1\leq i\leq k}{\text{span}}\eta_{i},\Phi \right).$$

As the basis set becomes increasingly rich, the right-hand side of the inequality converges to zero, implying that the lag-time-independent error must vanish.

To reduce the lag-time-dependent error, the best strategy is simply to increase the lag time. As *τ* → ∞, Theorem 3.4 guarantees that the lag-time-dependent error must decay exponentially quickly.

### 3.2. Estimation error.

In this subsection, we present formulas for the estimation error and explain how to calculate the mean squared estimation error using data.

#### 3.2.1. Formulas for the estimation error.

In applications of VAC, it is not typically possible to evaluate expectations *C*_*ij*_ (*τ*) = E_*μ*_ [*ϕ*_*i*_ (*X*_0_) *ϕ*_*j*_ (*X*_*τ*_)] exactly. Instead, trajectory data is used to provide estimates ${\hat{C}}_{ij}(\tau )\approx C_{ij}(\tau )$. In the asymptotic limit as $\hat{C}(\tau )\to C(\tau )$ and $\hat{C}(0)\to C(0)$, the estimation error is governed by the following asymptotic formulas.

Theorem 3.6 (estimation error). *Fix the basis set* Φ *and the lag time τ* > 0, *but allow matrices* $\hat{C}(0)$ *and* $\hat{C}(\tau )$ *to vary. Assume idealized VAC eigenfunctions are normalized so that* $\langle \gamma_{i}^{\tau },\gamma_{j}^{\tau }\rangle =\delta_{ij}$, *and recall that ν*_*i*_ (*τ*) *is the vector with* $\gamma_{i}^{\tau }=\sum_{j=1}^{n}v_{j}^{i}(\tau )\phi_{j}$. *Set*

(3.18) $${\hat{L}}_{ij}(\tau )=v^{i}{\left(\tau \right)}^{T}\left[\hat{C}(\tau )-\lambda_{j}^{\tau }\hat{C}(0)\right]v^{j}(\tau ),\;1\leq i,j\leq n.$$

*Then*, *VAC estimates have the following behavior as $\hat{C}(\tau )\to C(\tau )$ and $\hat{C}(0)\to C(0)$:*

1. *When there is a gap between* $\lambda_{k}^{\tau }$ *and all other $\lambda_{i}^{\tau }$ values, the kth VAC eigenvalue satisfies*
  (3.19) $${\hat{\lambda }}_{k}^{\tau }-\lambda_{k}^{\tau }={\hat{L}}_{kk}(\tau )+O\left(\|\hat{C}(\tau )-C(\tau ){\|}_{\text{F}}^{2}+\|\hat{C}(0)-C(0){\|}_{\text{F}}^{2}\right).$$
2. *When there is a gap between* $\left\{\lambda_{j}^{\tau },\ldots ,\lambda_{k}^{\tau }\right\}$ *and all other $\lambda_{i}^{\tau }$ values, the subspace* ${\text{span}}_{j\leq i\leq k}{\hat{\gamma }}_{i}^{\tau }$ *of VAC eigenfunctions satisfies*
  (3.20) $$d_{\text{F}}\left(\underset{j\leq i\leq k}{\text{span}}{\hat{\gamma }}_{i}^{\tau },\underset{j\leq i\leq k}{\text{span}}\gamma_{i}^{\tau }\right)={\left(\sum_{\begin{array}{c} l<j\\ or\;l>k \end{array}}\sum_{m=j}^{k}{\left|\frac{{\hat{L}}_{lm}^{\tau }}{\lambda_{l}^{\tau }-\lambda_{m}^{\tau }}\right|}^{2}\right)}^{1/2}+O\left(\|\hat{C}(\tau )-C(\tau ){\|}_{\text{F}}^{2}+\|\hat{C}(0)-C(0){\|}_{\text{F}}^{2}\right).$$
  *Moreover*, *the condition number for the subspace* ${\text{span}}_{j\leq i\leq k}{\hat{\gamma }}_{i}^{\tau }$ *is given by*
  (3.21) $$\underset{\begin{array}{c} \hat{C}(\tau )\to C(\tau )\\ \hat{C}(0)\to C(0) \end{array}}{\text{lim sup}}\frac{d_{\text{F}}\left(\underset{j\leq i\leq k}{\text{span}}{\hat{\gamma }}_{i}^{\tau },\underset{j\leq i\leq k}{\text{span}}\gamma_{i}^{\tau }\right)}{\|\hat{L}(\tau ){\|}_{\text{F}}}=\frac{1}{\text{min}\left\{\lambda_{j-1}^{\tau }-\lambda_{j}^{\tau },\lambda_{k}^{\tau }-\lambda_{k+1}^{\tau }\right\}},$$
  *with the conventions* $\lambda_{0}^{\tau }=\infty$ *and* $\lambda_{n+1}^{\tau }=-\infty$.

*Proof*. See subsection 5.5. ■

A useful quantity identified in Theorem 3.6 is the condition number (3.21), which quantifies VAC’s sensitivity to small errors in the matrices $\hat{C}(\tau )$ and $\hat{C}(0)$. In experiments, we find the condition number is a useful heuristic for judging whether a VAC estimation problem is easy or hard—more specifically, whether a large or small data set is required for accurate estimation. When the condition number for a subspace of VAC eigenfunctions is higher than 5 at all lag times, the numerical experiments in section 4 show that VAC is prone to experiencing large amounts of estimation error. Empirically, we can estimate the minimum condition number across all lag times by using

(3.22) $$\underset{\tau \geq 0}{\text{min}}\frac{1}{\text{min}\left\{{\hat{\lambda }}_{j-1}^{\tau }-{\hat{\lambda }}_{j}^{\tau },{\hat{\lambda }}_{k}^{\tau }-{\hat{\lambda }}_{k+1}^{\tau }\right\}}.$$

We recommend that VAC users identify the minimum condition number for various subspaces and focus on estimating the well-conditioned subspaces whenever possible. Additionally, we recommend that authors report the minimum condition number along with their VAC results, helping readers to assess whether the results could be affected by estimation error.

#### 3.2.2. Calculating the asymptotic mean squared estimation error using data.

Here, we explain how to calculate the mean squared estimation error using trajectory data. We assume for simplicity that the data consists of a single long stationary trajectory of the process *X*_*t*_. However, the estimation procedure described here could be generalized to other types of trajectory data.

Our approach for calculating the mean squared estimation error is based on the following convergence in distribution result.

Theorem 3.7. *Fix the basis set* Φ *and the lag time τ > 0, but allow the data set used in VAC to vary. Assume* E_*μ*_ |*ϕ*_*i*_ (*X*_0_)|^4^ < ∞ *for* 1 ≤ *i ≤ n. Assume that a stationary trajectory X*_0_, *X*_Δ_, *X*_2Δ_, …, *X*_*T*−Δ_ *is simulated and estimates ${\hat{C}}_{ij}(t)\approx C_{ij}(t)$ are formed using*

(3.23) $${\hat{C}}_{ij}(t)=\frac{\Delta }{T-t}\sum_{s=0}^{\frac{T-t}{\Delta }-1}\frac{\phi_{i}\left(X_{s\Delta }\right)\phi_{j}\left(X_{s\Delta +t}\right)+\phi_{j}\left(X_{s\Delta }\right)\phi_{i}\left(X_{s\Delta +t}\right)}{2}.$$

*Then, VAC estimates have the following behavior as T* → ∞:

1. *When there is a gap between* $\lambda_{k}^{\tau }$ *and all other $\lambda_{i}^{\tau }$ values, the kth VAC eigenvalue satisfies*
  (3.24) $$\sqrt{T}\left({\hat{\lambda }}_{k}^{\tau }-\lambda_{k}^{\tau }\right)\overset{D}{\to }Z_{kk}^{\tau }.$$
2. *When there is a gap between* $\left\{\lambda_{j}^{\tau },\ldots ,\lambda_{k}^{\tau }\right\}$ *and all other $\lambda_{i}^{\tau }$ values, the subspace* ${\text{span}}_{j\leq i\leq k}{\hat{\gamma }}_{i}^{\tau }$ *of VAC eigenfunctions satisfies*
  (3.25) $$Td_{\text{F}}{\left(\underset{j\leq i\leq k}{\text{span}}{\hat{\gamma }}_{i}^{\tau },\underset{j\leq i\leq k}{\text{span}}\gamma_{i}^{\tau }\right)}^{2}\overset{D}{\to }\sum_{\begin{array}{c} l<j\\ or\;l>k \end{array}}\sum_{m=j}^{k}{\left|\frac{Z_{lm}^{\tau }}{\lambda_{l}^{\tau }-\lambda_{m}^{\tau }}\right|}^{2}.$$
  *Here*, ${\left(Z_{lm}^{\tau }\right)}_{1\leq l,m\leq n}$ *is a mean-zero multivariate Gaussian random variable with variance terms*
  (3.26) $$\text{E}{\left|Z_{lm}^{\tau }\right|}^{2}=\Delta \sum_{s=-\infty }^{\infty }{\text{Cov}}_{\mu }\left[F_{lm}^{\tau }\left(X_{0},X_{\tau }\right),F_{lm}^{\tau }\left(X_{s\Delta },X_{s\Delta +\tau }\right)\right],$$
  *where we have defined*
  (3.27) $$F_{lm}^{\tau }(x,y)=\frac{\gamma_{l}^{\tau }(x)\gamma_{m}^{\tau }(y)+\gamma_{l}^{\tau }(y)\gamma_{m}^{\tau }(x)}{2}-\lambda_{m}^{\tau }\frac{\gamma_{l}^{\tau }(x)\gamma_{m}^{\tau }(x)+\gamma_{l}^{\tau }(y)\gamma_{m}^{\tau }(y)}{2}.$$

*Proof* See subsection 5.6. ■

The great value of Theorem 3.7 is that it suggests a data-driven approach for calculating the mean squared estimation error in the asymptotic limit as *T* → ∞. First, we can use the data set to estimate the $\text{E}{\left|Z_{lm}^{\tau }\right|}^{2}$ terms by means of (3.26). Second, we can substitute the $\text{E}{\left|Z_{lm}^{\tau }\right|}^{2}$ estimates into (3.24) or (3.25) to compute the mean squared estimation error for eigenvalues or invariant subspaces. In the accompanying supplemental material file SMM133598.pdf [local/web 301KB], we provide a step-by-step description of this estimation procedure.

In Figure 3, we calculate the mean squared estimation error by using a single trajectory of data. We find that it is possible to accurately identify the lag times at which the mean squared estimation error exceeds a critical threshold, such as 0.2. Moreover, in the numerical experiments in section 4, 0.2 is a typical level at which the estimation error begins to contribute significantly to VAC’s overall error. Therefore, in VAC applications we recommend calculating the mean squared error and avoiding lag times where the error exceeds such a threshold.

We conclude this section by considering three additional strategies to reduce the estimation error of VAC. The first strategy is to increase trajectory length. By increasing the length *T* of the trajectory, the estimation error consistently decreases at a $1/\sqrt{T}$ rate as shown in Figure 3.

The second strategy for reducing the estimation error is to prune the size of the basis set. We find in Theorem 3.6 that the squared estimation error increases linearly with the number of basis functions. Therefore, it is best to include only those basis functions that have the potential to overlap with the eigenfunctions of the transition operator.

The final strategy for reducing the estimation error is to select basis functions with favorable integrability properties. In Theorem 3.7, it is seen that the mean squared estimation error depends on the fourth moments of the idealized VAC coordinates. If the basis functions themselves have large kurtosis

(3.28) $$\frac{{\text{E}}_{\mu }{\left|\phi_{i}\left(X_{0}\right)-{\text{E}}_{\mu }\left[\phi_{i}\left(X_{0}\right)\right]\right|}^{4}}{{\left({\text{E}}_{\mu }{\left|\phi_{i}\left(X_{0}\right)-{\text{E}}_{\mu }\left[\phi_{i}\left(X_{0}\right)\right]\right|}^{2}\right)}^{2}},$$

this can increase the estimation error in VAC calculations. Favorable integrability properties may be one factor that helps explain the success of Markov state models, in which the basis consists of indicator functions on a partition of the state space. The fourth moments of indicator functions are often well controlled, compared to, e.g., higher-order polynomials of the coordinate axes.

## 4. Numerical experiments.

In this section, we report on two numerical experiments that illustrate the major factors impacting VAC accuracy. These experiments show how computing the VAC condition number and the mean squared estimation error can help to improve VAC’s results.

### 4.1. Varying the basis size and trajectory length.

First, we apply VAC to estimate the span of eigenfunctions *η*_1_, *η*_2_, and *η*_3_ for the Ornstein–Uhlenbeck (OU) process

(4.1) $$dX=-X\;dt+\sqrt{2}\;dW.$$

In two different trials, we show how VAC’s accuracy depends on the size of the basis set and the length of the simulated trajectory. The number of basis functions and the trajectory length are varied as follows:

**Table:** 

	Trial 1	Trial 2
Basis functions	*n* = 20	*n* = 50
Trajectory length	*T* = 10^4^	*T* = 500

In both trials, the basis functions are indicator functions on disjoint intervals.

**Figure 4.**
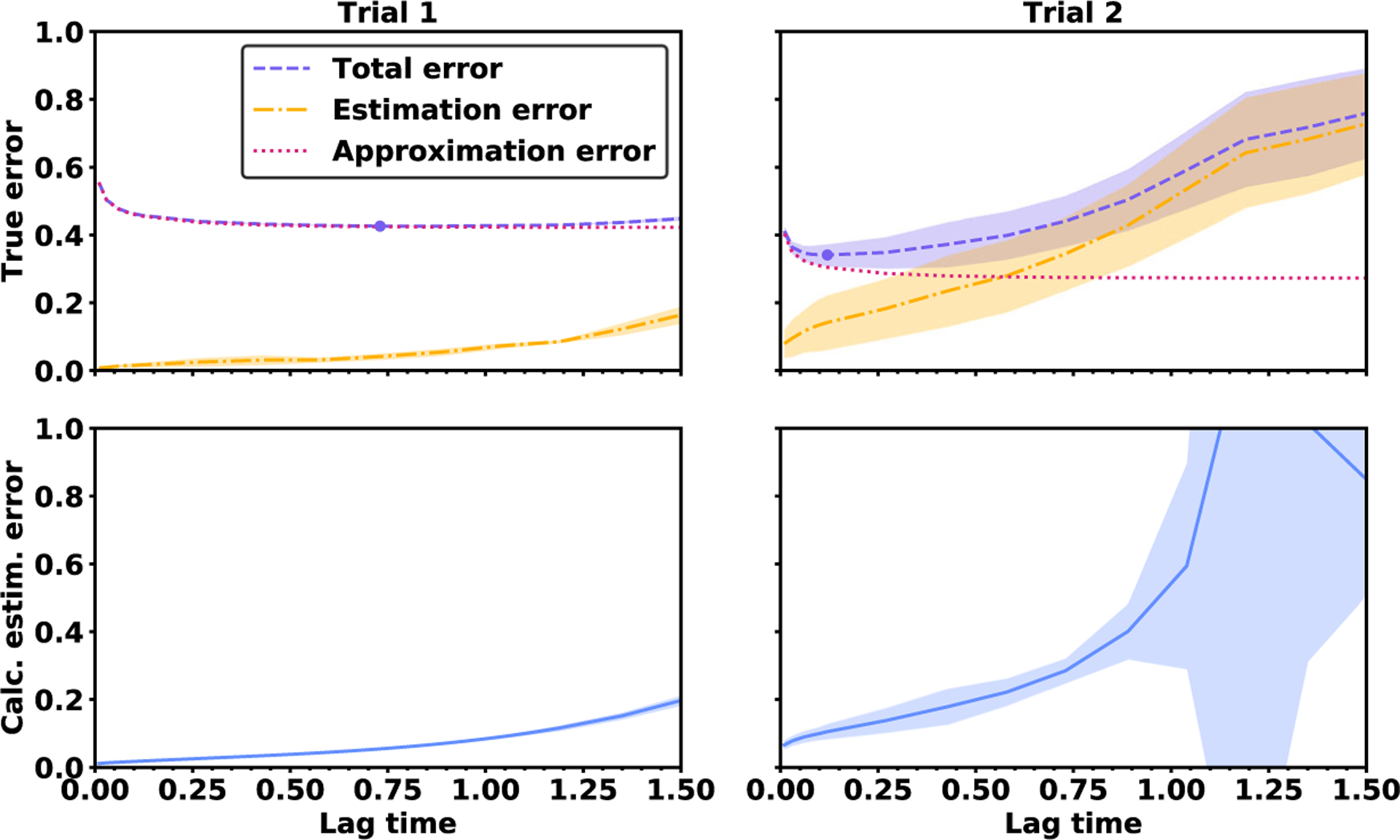
True error and calculated error for the OU process. Top: The bold line indicates the true root mean squared error over 30 independent trajectories, while the shaded region indicates the mean ± 1 standard deviation. The purple dot shows the optimal lag time. Bottom: The calculated root mean squared estimation error obtained from each of the 30 trajectories.

The two different trials demonstrate that the breakdown of approximation error and estimation error is sensitive to context, as seen in Figure 4. The approximation error is higher in trial 1 because of the smaller set of basis functions, whereas the estimation error is higher in trial 2 because of the smaller data set. In trial 1, it is optimal to use a comparatively long lag time of *τ* = 0.7 to reduce the approximation error. In contrast, in trial 2 it is optimal to use a comparatively short lag time of *τ* = 0.1 to avoid the increase in estimation error at longer lag times.

In addition to showing the true error levels, Figure 4 shows the root mean squared estimation error calculated directly from the data. In trial 1, the calculated estimation error remains below 0.2 for lag times up to 1.5. Therefore, the VAC practitioner can infer that the data set is rich enough to take high lag times without experiencing large estimation error. However, in trial 2, the calculated estimation rises more rapidly, reaching a level of 0.2 when the lag time is just *τ* ≈ 0.5. In this case, the VAC practitioner can infer that the data set is not rich enough to take *τ* > 0.5.

An alternative lag time selection strategy called *implied timescale analysis* has been advocated in the past by VAC researchers [45]. In this strategy, the VAC eigenvalues are used to compute the implied timescales

(4.2) $$\frac{-\tau }{\text{log}\left({\hat{\lambda }}_{i}^{\tau }\right)}.$$

If VAC eigenvalues were perfect estimates of the true eigenvalues, then implied timescales would be perfectly flat and they would equal $\sigma_{i}^{-1}$. In practice, however, implied timescales are not flat. They increase quickly at short lag times and then increase more slowly at long lag times. To cut down on VAC’s error, Swope and coauthors [45] proposed selecting a long enough lag time so that the implied timescales for the eigenfunctions of interest are approximately level.

**Figure 5.**
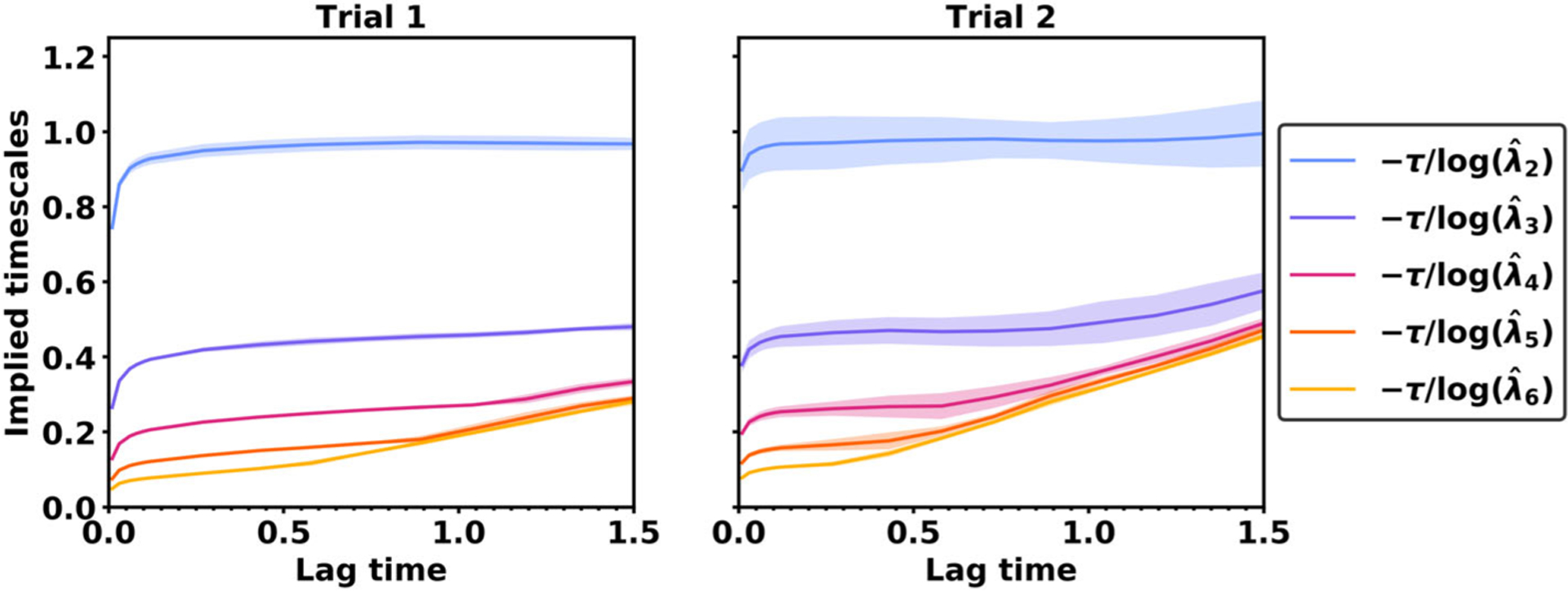
Implied timescales of the OU process.

Figure 5 presents the implied timescales for the OU process. From the figure it is clear that the implied timescales cannot be used to assess the estimation error. The estimation error is much higher in the second trial, yet the implied timescales for trial 1 and trial 2 are similar. However, implied timescales may help assess the approximation error. As the lag time is increased from *τ* = 0 to *τ* = 0.1, the second and third implied timescales become much flatter, which provides an accurate indication that the approximation error is decreasing and beginning to settle.

We conclude that implied timescale analysis may provide an approach for assessing approximation error that is complementary to our approach for assessing the estimation error. Whereas our approach is useful for identifying and avoiding the error that is prevalent at long lag times, implied timescale analysis may be useful for identifying and avoiding the error that is prevalent at short lag times. However, while our approach for computing the mean squared estimation error is rigorously justified, it remains an open research problem to rigorously justify this proposed relationship between the implied timescales and the approximation error.

### 4.2. Varying the size of the subspace.

In our second experiment, we apply VAC to estimate the eigenfunctions of the diffusion process

(4.3) $$dX=-\frac{1}{2}\sigma \sigma^{T}\nabla U(X)dt+\sigma dW,$$

where the potential *U* and the diffusion matrix *σ* are given by

(4.4) $$U\left(x_{1},x_{2}\right)=4x_{1}^{4}-8x_{1}^{2}+x_{1}+0.5x_{2}^{2},\;\sigma =\left(\begin{array}{cc} 2 & 0\\ -1 & \sqrt{3} \end{array}\right).$$

We simulate a stationary trajectory of length *T* = 500 and then apply VAC using the basis $\text{set }\left\{1,x_{1},x_{2},x_{1}^{2},x_{1}x_{2},x_{2}^{2}\right\}$.

**Figure 6.**
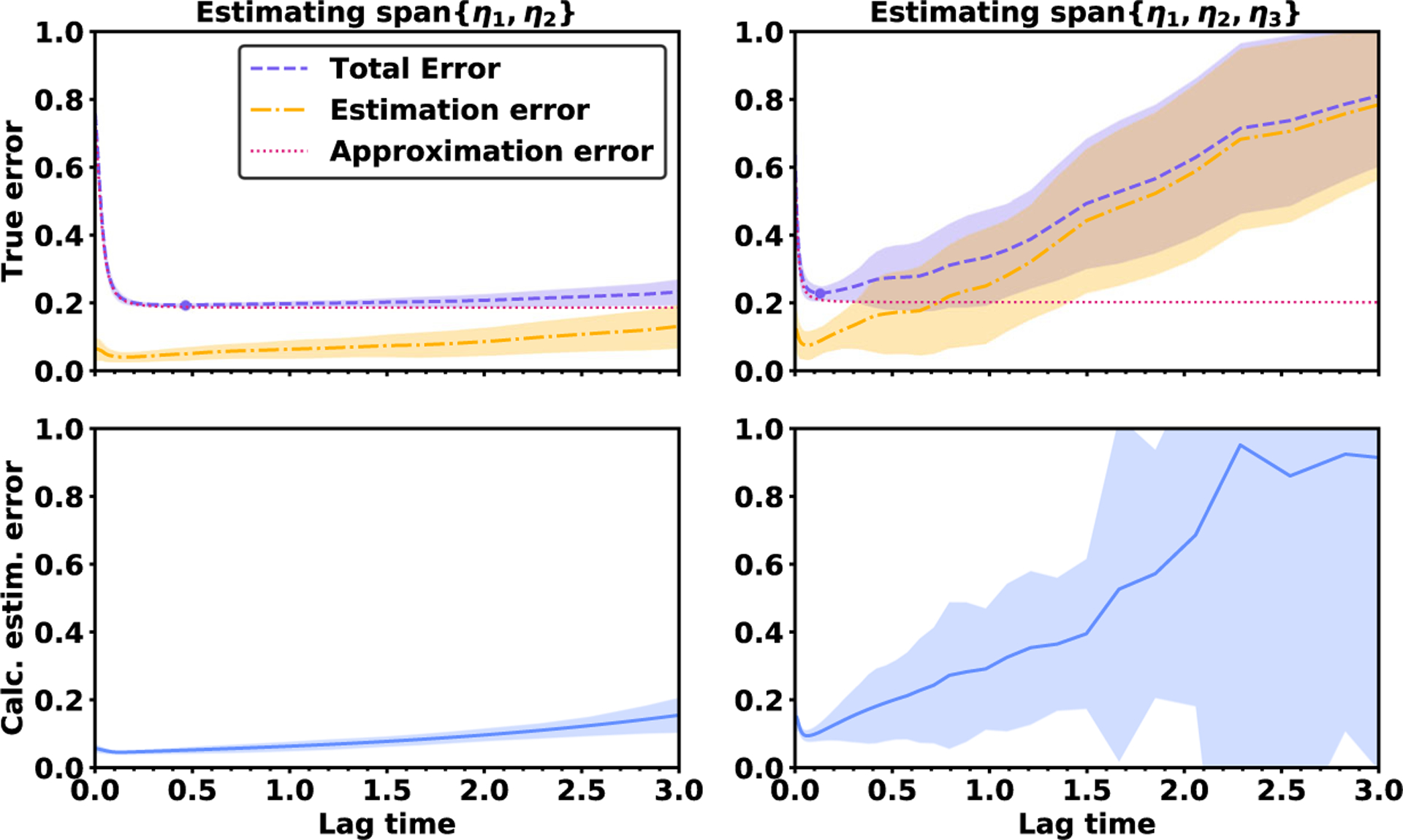
When the minimum condition number is 2.0, the estimation error is low (left). When the minimum condition number is 9.5, the estimation error is much higher (right).

We investigate how the accuracy changes when VAC is used to estimate two subspaces of different sizes: span {*η*_1_, *η*_2_} and span {*η*_1_, *η*_2_, *η*_3_}. When estimating span {*η*_1_, *η*_2_}, there is a wide range of lag times that all lead to low error levels. As seen in Figure 6, the total error decreases between lag times of *τ* = 0 and *τ* = 0.2, but it is nearly constant for all lag times between *τ* = 0.2 and *τ* = 1.5. In contrast, when estimating span {*η*_1_, *η*_2_, *η*_3_}, the total error is V-shaped with a distinct minimum at the lag time *τ* = 0.2. The error rises rapidly as the lag time is increased beyond *τ* = 0.2 due to an upsurge in the estimation error.

What explains the different error profiles when estimating the subspace span {*η*_1_, *η*_2_} versus span {*η*_1_, *η*_2_, *η*_3_}? The explanation is not a difference in the data set or the basis set, since these factors remain the same when estimating the two subspaces. Rather, the increase in estimation error is due to the much higher condition number for the subspace span {*η*_1_, *η*_2_, *η*_3_}.*·* No matter how the lag time is selected, the inverse spectral gap ${\left(\lambda_{4}^{\tau }-\lambda_{3}^{\tau }\right)}^{-1}$ is at least as high as 9.5. In contrast, when estimating the subspace span {*η*_1_, *η*_2_}, the minimum condition number ${\text{min}}_{\tau }{\left(\lambda_{3}^{\tau }-\lambda_{2}^{\tau }\right)}^{-1}$ is just 2.0. Here we see that a high condition number is associated with increased levels of estimation error and a stronger relationship between estimation error and lag time.

**Figure 7.**
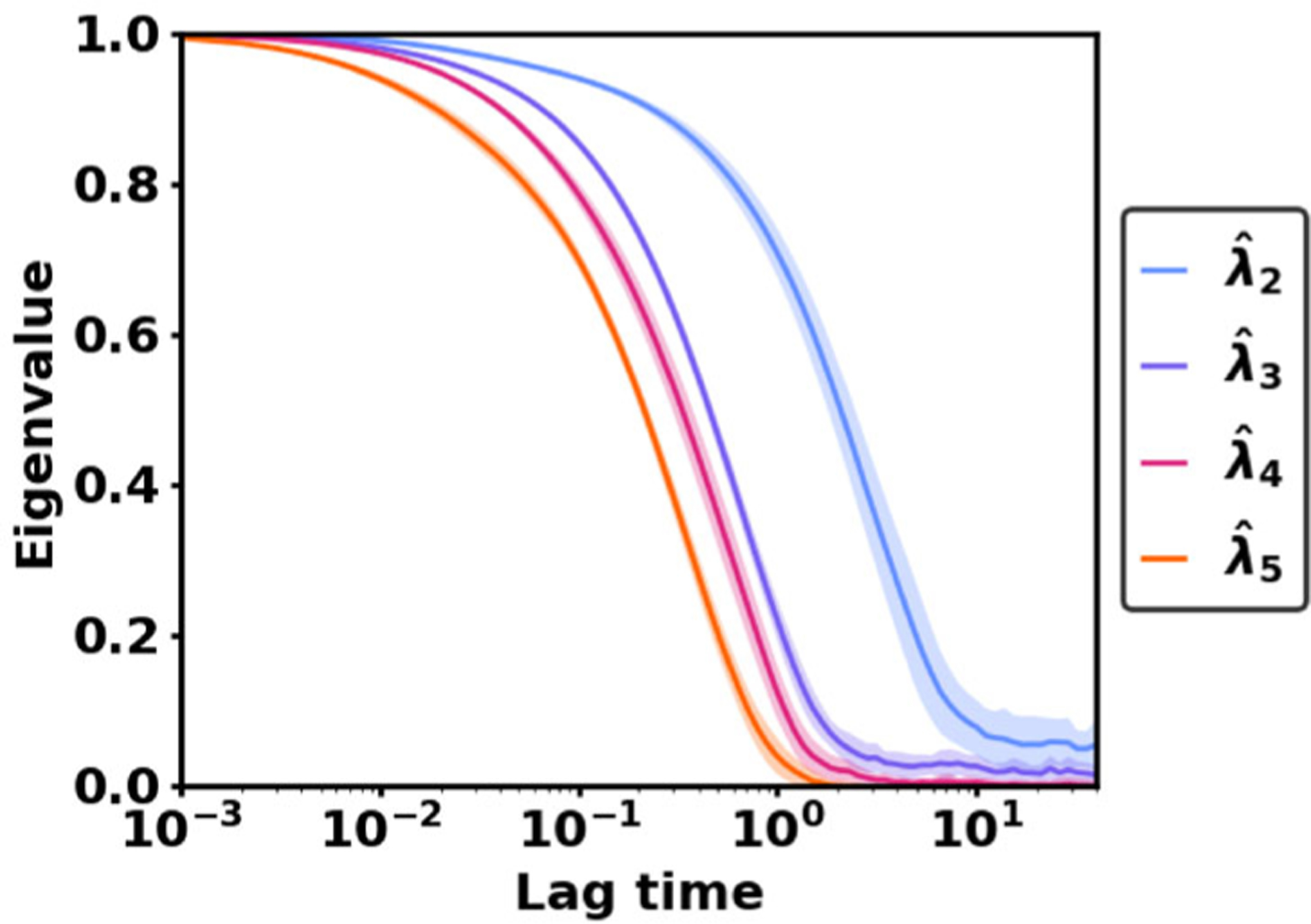
VAC eigenvalues.

To avoid situations where the estimation error is uncontrollably high, VAC users should identify well-conditioned subspaces and focus on estimating these subspaces whenever possible. As shown in the VAC eigenvalue plot in Figure 7, eigenvalues for well-conditioned subspaces visually stand apart from the rest of the eigenvalues. The large gap between the second and third VAC eigenvalues indicates a natural separation in timescales, which implies that span {*η*_1_, *η*_2_} is a well-conditioned subspace.

## 5. Mathematical derivations.

In this section, we prove the mathematical results presented in Theorem 3.4, Theorem 3.5, Theorem 3.6, and Theorem 3.7.

### 5.1. Building mathematical intuition.

Before proving Theorem 3.4, we identify the intuitive mathematical reason why idealized VAC estimates converge at long lag times. Applying the spectral decomposition (2.13), we find that each matrix entry *C*_*ij*_ (*τ*) has an exponentially decaying structure.

(5.1) $$C_{ij}(\tau )=\langle \phi_{i},T_{\tau }\phi_{j}\rangle =\sum_{l=1}^{r}e^{-\sigma_{l}\tau }\langle \eta_{l},\phi_{i}\rangle \langle \eta_{l},\phi_{j}\rangle +O\left(e^{-\sigma_{r+1}\tau }\right),\;\tau \to \infty .$$

Thus, the matrix *C* (*τ*) is the sum of exponentially decaying rank-one matrices

(5.2) $$C(\tau )=\sum_{l=1}^{r}e^{-\sigma_{l}\tau }\langle \eta_{l},\vec{\phi }\rangle {\langle \eta_{l},\vec{\phi }\rangle }^{T}+O\left(e^{-\sigma_{r+1}\tau }\right),\;\tau \to \infty ,$$

where we have used the shorthand $\langle \eta_{l},\vec{\phi }\rangle$ to denote the vector with entries 〈η_*l*_, *ϕ*_*i*_〉.

To approximate the behavior of idealized VAC at long lag times, we remove the smallest terms in the expansion (5.2) and replace *C* (*τ*) by the sum of *k* rank-one matrices

(5.3) $$\sum_{l=1}^{k}e^{-\sigma_{l}\tau }\langle \eta_{l},\vec{\phi }\rangle {\langle \eta_{l},\vec{\phi }\rangle }^{T}.$$

When the rank-*k* approximation is used in place of *C* (*τ*), it results that the top *k* idealized VAC eigenfunctions $\gamma_{1}^{\tau },\ldots ,\gamma_{k}^{\tau }$ span the subspace

(5.4) $$\underset{1\leq i\leq k}{\text{span}}\;q_{i}=P_{\Phi }\underset{1\leq i\leq k}{\text{span}}\eta_{i}.$$

Therefore, the truncation argument helps intuitively explain the convergence of idealized eigenfunctions $\gamma_{i}^{\tau }$ to orthogonalized projections *q*_*i*_ as *τ* → ∞. Our proofs in subsection 5.2, subsection 5.3, and subsection 5.4 essentially serve to justify the truncation argument and to provide rigorous bounds on the convergence.

### 5.2. Convergence of eigenvalues.

In this section, we verify the statement in Theorem 3.4 that the *k*th idealized eigenvalue converges

(3.12) $$\frac{\lambda_{k}^{\tau }}{e^{-\sigma_{k}\tau }}\to {\langle \eta_{k},q_{k}\rangle }^{2}$$

in the limit *τ* → ∞, provided there is a gap between *σ*_*k*_ and all other *σ*_*i*_ values. To prove this result, our main tool is the min-max principle for self-adjoint operators [36].

Lemma 5.1. *Consider a quasi-compact self-adjoint operator*

(5.5) $$A=\sum_{i=1}^{r}\lambda_{i}P_{\eta_{i}}+R.$$

*Here, η*_1_, *η*_2_, … , *η*_*r*_ *are orthonormal eigenfunctions of A with eigenvalues λ*_1_ ≥ *λ*_2_ ≥ ⋯ ≥ *λ*_*r*_, *and the spectrum of R lies in* (−∞, *λ*_*r*_). *Then, for each* 1 ≤ *k* ≤ *r*,

(5.6) $$\lambda_{k}(A)=\underset{\text{dim}(\text{H})=k}{\text{max}}\underset{\eta \in \text{H}}{\text{min}}\frac{\langle \eta ,A\eta \rangle }{\langle \eta ,\eta \rangle }.$$

Before applying the min-max principle, we derive two estimates.

Proposition 5.2. *For any ϕ* ∈ Φ ∩ (span_1≤*i*≤*k*−1_ *q*_*i*_)^⊥^,

(5.7) $$\frac{\langle \phi ,T_{\tau }\phi \rangle }{\langle \phi ,\phi \rangle }\leq e^{-\sigma_{k}\tau }{\langle \eta_{k},q_{k}\rangle }^{2}+e^{-\sigma_{k+1}\tau }.$$

*Proof* Calculate

$$\begin{array}{lll} \left(5.8\right) & \langle \phi ,T_{\tau }\phi \rangle & =\langle \phi ,\left(\sum_{i=k}^{r}e^{-\sigma_{i}\tau }P_{\eta_{i}}+R_{\tau }\right)\phi \rangle \\ \left(5.9\right) & & =e^{-\sigma_{k}\tau }{\langle \eta_{k},\phi \rangle }^{2}+\langle \phi ,\left(\sum_{i=k+1}^{r}e^{-\sigma_{i}\tau }P_{\eta_{i}}+R_{\tau }\right)\phi \rangle \\ \left(5.10\right) & & \leq e^{-\sigma_{k}\tau }{\langle \eta_{k},q_{k}\rangle }^{2}{\langle q_{k},\phi \rangle }^{2}+e^{-\sigma_{k+1}\tau }\langle \phi ,\phi \rangle \\ \left(5.11\right) & & \leq e^{-\sigma_{k}\tau }{\langle \eta_{k},q_{k}\rangle }^{2}\langle \phi ,\phi \rangle +e^{-\sigma_{k+1}\tau }\langle \phi ,\phi \rangle . \end{array}$$

■

Proposition 5.3. *Set* H_1:*k*−1_ = span_1≤*i*≤*k*−1_ *η*_*i*_. *Then for any q* ∈ *Q*_1:*k*_ = span_1≤*i*≤*k*_ *q*_*i*_,

(5.12) $$\frac{\langle q,T_{\tau }q\rangle }{\langle q,q\rangle }\geq e^{-\sigma_{k}\tau }\left({\langle \eta_{k},q_{k}\rangle }^{2}-\frac{1}{e^{\left(\sigma_{k}-\sigma_{k-1}\right)\tau }\left(1-d_{2}^{2}\left({\text{H}}_{1:k-1},\Phi \right)\right)-{\langle \eta_{k},q_{k}\rangle }^{2}}\right),$$

*provided the denominator term is positive*.

*Proof*. It suffices to consider the ∥*q*∥ = 1 case. Then, *q* can be decomposed as *q = aq*′+*bq*_*k*_, where *a*^*2*^ + *b*^*2*^ = 1, *q*′ ∈ *Q*_1:*k*−1_, and ∥*q*′∥ = 1. It follows that

$$\begin{array}{lll} \left(5.13\right) & \langle q,T_{\tau }q\rangle & \geq \langle q,\left(e^{-\sigma_{k-1}\tau }P_{{\text{H}}_{1:k-1}}+e^{-\sigma_{k}\tau }P_{\eta_{k}}\right)q\rangle \\ \left(5.14\right) & & =a^{2}e^{-\sigma_{k-1}\tau }{\left\|P_{{\text{H}}_{1:k-1}}q^{\prime }\right\|}^{2}+e^{-\sigma_{k}\tau }{\langle \eta_{k},aq^{\prime }+bq_{k}\rangle }^{2}. \end{array}$$

Thus, 〈*q*, *T*_*τ*_*q*〉 is bounded from below by the lowest eigenvalue of

(5.15) $$M=e^{-\sigma_{k-1}\tau }{\left\|P_{{\text{H}}_{1:k-1}}q^{\prime }\right\|}^{2}\left(\begin{array}{cc} 1 & 0\\ 0 & 0 \end{array}\right)+e^{-\sigma_{k}\tau }\left(\begin{array}{cc} {\langle \eta_{k},q^{\prime }\rangle }^{2} & \langle \eta_{k},q^{\prime }\rangle \langle \eta_{k},q_{k}\rangle \\ \langle \eta_{k},q^{\prime }\rangle \langle \eta_{k},q_{k}\rangle & {\langle \eta_{k},q_{k}\rangle }^{2} \end{array}\right).$$

For any symmetric real-valued matrix $M=\left(\begin{array}{ll} a & b\\ b & c \end{array}\right)$ with *a* > *c*, the lowest eigenvalue is at least as large as *c* − *b*^2^*/* (*a* − *c*) [22], We can check that

(5.16) $${\left\|P_{{\text{H}}_{1:k-1}}q^{\prime }\right\|}^{2}=1-{\left\|P_{{\text{H}}_{1:k-1}^{⊥}}q^{\prime }\right\|}^{2}\geq 1-d_{2}^{2}\left({\text{H}}_{1:k-1},\Phi \right).$$

Therefore, the lowest eigenvalue of the matrix *M* is at least as large as

(5.17) $$e^{-\sigma_{k}\tau }{\langle \eta_{k},q_{k}\rangle }^{2}-\frac{e^{-2\sigma_{k}\tau }}{e^{-\sigma_{k-1}\tau }\left(1-d_{2}^{2}\left({\text{H}}_{1:k-1},\Phi \right)\right)-e^{-\sigma_{k}\tau }{\langle \eta_{k},q_{k}\rangle }^{2}}.$$

■

*Proof of* (3.12). Using the min-max principle and Proposition 5.2, we obtain

(5.18) $$\lambda_{k}^{\tau }=\underset{\text{dim}(\text{H})=k,\text{H}\subseteq \Phi }{\text{max}}\underset{\eta \in \text{H}}{\text{min}}\frac{\langle \eta ,T_{\tau }\eta \rangle }{\langle \eta ,\eta \rangle }\leq e^{-\sigma_{k}\tau }{\langle \eta_{k},q_{k}\rangle }^{2}(1+o(1)).$$

as *τ* →∞. Using the min-max principle and Proposition 5.3, we obtain

(5.19) $$\lambda_{k}^{\tau }=\underset{\text{dim}(\text{H})=k,\text{H}\subseteq \Phi }{\text{max}}\underset{\eta \in \text{H}}{\text{min}}\frac{\langle \eta ,T_{\tau }\eta \rangle }{\langle \eta ,\eta \rangle }\geq e^{-\sigma_{k}\tau }{\langle \eta_{k},q_{k}\rangle }^{2}(1+o(1)).$$

■

### 5.3. Convergence of invariant subspaces.

In this section, we verify the statement in Theorem 3.4 that the subspace ${\text{span}}_{j\leq i\leq k}\gamma_{i}^{\tau }$ of idealized VAC eigenfunctions converges as

(3.13) $$\underset{j\leq i\leq k}{\text{span}}\gamma_{i}^{\tau }\to \underset{j\leq i\leq k}{\text{span}}q_{i}$$

in the limit *τ* →∞, provided there is a gap between {*σ*_*j*_, … , *σ*_*k*_} and all other *σ*_*i*_ values. To prove this result, our main tool is a well-known lemma due to Davis and Kahan [6].

Lemma 5.4. *Suppose A and B are self-adjoint operators and U and W are closed subspaces. If the spectrum of ${P_{U}A|}_{U}$ lies in the interval* [*a*, *b*] *and the spectrum of* ${P_{W}B|}_{W}$ *lies in* (−∞, *a* – *δ*] ∪ [*b* + *δ*, ∞), *then*

(5.20) $$\delta {\left\|P_{W}P_{U}\right\|}_{\text{F}}\leq {\left\|P_{W}P_{U}AP_{U}-P_{W}BP_{W}P_{U}\right\|}_{\text{F}}.$$

The Davis and Kahan lemma leads to the following error bound.

Proposition 5.5. *When* $\lambda_{k}^{\tau }>e^{-\sigma_{k+1}}$, *the distance between subspaces* $\Gamma_{1:k}^{\tau }={\text{span}}_{1\leq i\leq k}\gamma_{i}^{\tau }$ *and Q*_1:k_ = span_1≤*i*≤*k*_ *q*_*i*_ *is bounded by*

(5.21) $$d_{\text{F}}\left(\Gamma_{1:k}^{\tau },Q_{1:k}\right)\leq \frac{e^{-\sigma_{k+1}\tau }}{2\left(\lambda_{k}^{\tau }-e^{-\sigma_{k+1}\tau }\right)}d_{\text{F}}\left(\underset{1\leq i\leq k}{\text{span}}\eta_{i},\Phi \right).$$

*Proof*. The spectrum of ${P_{\Phi \cap Q_{1:k}^{⊥}}T_{\tau }|}_{\Phi \cap Q_{1:k}^{⊥}}$ lies in the interval $\left[0,e^{-\sigma_{k+1}\tau }\right]$, while the spectrum of ${P_{\Gamma_{1:k}^{\tau }}T_{\tau }|}_{\Gamma_{1:k}^{\tau }}$ lies in the interval $\left[\lambda_{k}^{\tau },1\right]$. Therefore, the spectral gap is at least $\lambda_{k}^{\tau }-e^{-\sigma_{k+1}\tau }$. We calculate

$$\begin{array}{lll} \left(5.22\right) & \left(\lambda_{k}^{\tau }-e^{-\sigma_{k+1}\tau }\right){\left\|P_{\Phi \cap Q_{1:k}^{⊥}}P_{\Gamma_{1:k}^{\tau }}\right\|}_{F} & \leq {\left\|P_{\Phi \cap Q_{1:k}^{⊥}}P_{\Gamma_{1:k}^{\tau }}T_{\tau }P_{\Gamma_{1:k}^{\tau }}-P_{\Phi \cap Q_{1:k}^{⊥}}T_{\tau }P_{\Phi \cap Q_{1:k}^{⊥}}P_{\Gamma_{1:k}^{\tau }}\right\|}_{\text{F}}\\ \left(5.23\right) & & ={\left\|P_{\Phi \cap Q_{1:k}^{⊥}}T_{\tau }P_{\Gamma_{1:k}^{\tau }}-P_{\Phi \cap Q_{1:k}^{⊥}}T_{\tau }P_{\Phi \cap Q_{1:k}^{⊥}}P_{\Gamma_{1:k}^{\tau }}\right\|}_{F}\\ \left(5.24\right) & & ={\left\|P_{\Phi \cap Q_{1:k}^{⊥}}T_{\tau }P_{Q_{1:k}}P_{\Gamma_{1:k}^{\tau }}\right\|}_{\text{F}}\\ \left(5.25\right) & & \leq {\left\|P_{\Phi \cap Q_{1:k}^{⊥}}T_{\tau }P_{Q_{1:k}}\right\|}_{\text{F}}, \end{array}$$

where we have used the fact that $\Gamma_{1:k}^{\tau }$ is an invariant subspace of *P*_Φ_*T*_*τ*_*P*_Φ_. Next, we introduce the subspace H_1:*k*_ = span_1≤*i*≤*k*_ *η*_*i*_, which is orthogonal to $\Phi \cap Q_{1:k}^{⊥}$ Then,

(5.26) $${\left\|P_{{\text{H}}_{1:k}^{⊥}}P_{Q_{1:k}}\right\|}_{\text{F}}={\left\|P_{{\text{H}}_{1:k}}P_{Q_{1:k}^{⊥}}\right\|}_{\text{F}}={\left\|P_{{\text{H}}_{1:k}}P_{\Phi^{⊥}}\right\|}_{\text{F}}=d_{\text{F}}\left(\underset{1\leq i\leq k}{\text{span}}\eta_{i},\Phi \right).$$

To complete the theorem, it is enough to show that

(5.27) $${\left\|P_{\Phi \cap Q_{1:k}^{⊥}}T_{\tau }P_{Q_{1:k}}\right\|}_{\text{F}}\leq \frac{e^{-\sigma_{k+1}\tau }}{2}{\left\|P_{{\text{H}}_{1:k}^{⊥}}P_{Q_{1:k}}\right\|}_{\text{F}}.$$

To prove (5.27), we apply a useful property of the Frobenius norm. For bounded linear operators *A* and *B*, if it is true that ∥*Au* ∥ ≤∥*Bu*∥ for all *u*, then it follows that ∥*A*∥_F_ ≤ ∥*B*∥_F_ [12], Using this property, it is sufficient to prove that

(5.28) $$\left\|P_{\Phi \cap Q_{1:k}^{⊥}}T_{\tau }q\right\|\leq \frac{e^{-\sigma_{k+1}\tau }}{2}\left\|P_{{\text{H}}_{1:k}^{⊥}}q\right\|,\;q\in Q_{1:k}.$$

Moreover, it is sufficient to prove that

(5.29) $$\left|\langle \phi ,T_{t}q\rangle \right|\leq \frac{e^{-\sigma_{k+1}\tau }}{2}$$

for all $\phi \in \Phi \cap Q_{1:k}^{⊥}$ and *q* ∈ *Q*_1:*k*_ with $\|\phi \|=\left\|P_{{\text{H}}_{1:k}^{⊥}}q\right\|=1$. We observe that

(5.30) $${\left\|P_{{\text{H}}_{1:k}^{⊥}}(\phi \pm q)\right\|}^{2}={\left\|\phi \pm P_{{\text{H}}_{1:k}^{⊥}}q\right\|}^{2}=2\pm 2\langle \phi ,P_{{\text{H}}_{1:k}^{⊥}}q\rangle =2\pm 2\langle \phi ,q\rangle =2.$$

Using the polarization identity and the fact that ${\text{H}}_{1:k}^{⊥}$ is an invariant subspace of *T*_*τ*_, we conclude that

$$\begin{array}{lll} \left(5.31\right) & \left|\langle \phi ,T_{\tau }q\rangle \right| & =\left|\langle P_{{\text{H}}_{1:k}^{⊥}}\phi ,T_{\tau }P_{{\text{H}}_{1:k}^{⊥}}q\rangle \right|\\ \left(5.32\right) & & =\left|\frac{1}{4}\langle P_{{\text{H}}_{1:k}^{⊥}}(\phi +q),T_{\tau }P_{{\text{H}}_{1:k}^{⊥}}(\phi +q)\rangle -\frac{1}{4}\langle P_{{\text{H}}_{1:k}^{⊥}}(\phi -q),T_{\tau }P_{{\text{H}}_{1:k}^{⊥}}(\phi -q)\rangle \right|\\ \left(5.33\right) & & \leq \frac{1}{2}{\left\|P_{{\text{H}}_{1:k}^{⊥}}T_{\tau }P_{{\text{H}}_{1:k}^{⊥}}\right\|}_{2}\\ \left(5.34\right) & & \leq \frac{e^{-\sigma_{k+1}\tau }}{2}. \end{array}$$

■

*Proof of Theorem* 3.5. When $\lambda_{k}^{\tau }>e^{-\sigma_{k+1}}$, Proposition 5.5 allows us to calculate

$$\begin{array}{lll} \left(5.35\right) & d_{\text{F}}^{2}\left(\underset{1\leq i\leq k}{\text{span}}\eta_{i},\Phi \right) & \leq d_{\text{F}}^{2}\left(\underset{1\leq i\leq k}{\text{span}}\gamma_{i}^{\tau },\underset{1\leq i\leq k}{\text{span}}\eta_{i}\right)\\ \left(5.36\right) & & ={\left\|P_{\Phi^{⊥}}P_{{\text{H}}_{1:k}}\right\|}_{\text{F}}^{2}+{\left\|P_{{\left(\Gamma_{1:k}^{\tau }\right)}^{⊥}}P_{\Phi }P_{{\text{H}}_{1:k}}\right\|}_{\text{F}}^{2}\\ \left(5.37\right) & & ={\left\|P_{\Phi ⊥}P_{{\text{H}}_{1:k}}\right\|}_{\text{F}}^{2}+{\left\|P_{{\left(\Gamma_{1:k}^{\tau }\right)}^{⊥}}P_{Q_{1:k}}P_{{\text{H}}_{1:k}}\right\|}_{\text{F}}^{2}\\ \left(5.38\right) & & \leq \left(1+\frac{1}{4}{\left|\frac{e^{-\sigma_{k+1}\tau }}{\lambda_{k}^{\tau }-e^{-\sigma_{k+1}\tau }}\right|}^{2}\right)d_{\text{F}}^{2}\left(\underset{1\leq i\leq k}{\text{span}}\eta_{i},\Phi \right). \end{array}$$

■

*Proof of* (3.13). When there is a gap between {*σ*_*j*_, … , *σ*_*k*_} and all other σ_*i*_ values, Proposition 5.5 shows that ${\text{span}}_{1\leq i\leq j}\gamma_{i}^{\tau }\to {\text{span}}_{1\leq i\leq k}q_{i}$ and ${\text{span}}_{1\leq i\leq k}\gamma_{i}^{\tau }\to {\text{span}}_{1\leq i\leq k}q_{i}$ in the limit *τ* → ∞. By applying Lemma 2.5, we verify ${\text{span}}_{j\leq i\leq k}\gamma_{i}^{\tau }\to {\text{span}}_{j\leq i\leq k}q_{i}$. ■

### 5.4. Exponential speed of convergence.

In this section, we verify the last statement in Theorem 3.4 that the top *k* idealized VAC eigenfunctions satisfy

(3.14) $$d_{\text{F}}\left(\underset{1\leq i\leq k}{\text{span}}\gamma_{i}^{\tau },\underset{1\leq i\leq k}{\text{span}}q_{i}\right)\frac{\lambda_{k}^{\tau }}{\lambda_{k+1}^{\tau }}\to \left|\frac{\langle \eta_{k+1},q_{k}\rangle }{\langle \eta_{k+1},q_{k+1}\rangle }\right|$$

in the limit *τ* → ∞, provided there is a gap between *σ*_*k*_ and all other *σ*_*i*_ values and a gap between *σ*_*k*+1_ and all other *σ*_*i*_ values.

*Proof of* (3.14). If there are fewer orthogonalized projection functions ${\left(q_{i}\right)}_{1\leq i\leq p}$ compared to basis functions ${\left(\phi_{i}\right)}_{1\leq i\leq n}$, we select additional functions ${\left(q_{i}\right)}_{p+1\leq i\leq n}$ so that ${\left(q_{i}\right)}_{1\leq i\leq n}$ is a complete orthonormal basis for Φ. Then,

$$\begin{array}{lll} \left(5.39\right) & d_{\text{F}}\left(\underset{1\leq i\leq k}{\text{span}}\gamma_{i}^{\tau },\underset{1\leq i\leq k}{\text{span}}q_{i}\right) & ={\left\|\left(\sum_{i=k+1}^{n}q_{i}\langle q_{i},\cdot \rangle \right)\left(\sum_{j=1}^{k}\gamma_{j}^{\tau }\langle \gamma_{j}^{\tau },\cdot \rangle \right)\right\|}_{F}\\ \left(5.40\right) & & ={\left\|\sum_{i=k+1}^{n}\sum_{j=1}^{k}q_{i}\langle q_{i},\gamma_{j}^{\tau }\rangle \langle \gamma_{i}^{\tau },\cdot \rangle \right\|}_{F}\\ \left(5.41\right) & & ={\left(\sum_{i=k+1}^{n}\sum_{j=1}^{k}{\langle q_{i},\gamma_{j}^{\tau }\rangle }^{2}\right)}^{1/2}. \end{array}$$

The terms $\langle q_{i},\gamma_{j}^{\tau }\rangle$ are determined by the eigenvalue equation

(5.42) $$\lambda_{j}^{\tau }\langle q_{i},\gamma_{j}^{\tau }\rangle =\langle q_{i},T_{\tau }\gamma_{j}^{\tau }\rangle =\sum_{l=1}^{n}\langle q_{i},T_{\tau }q_{l}\rangle \langle q_{l},\gamma_{j}^{\tau }\rangle .$$

As *τ* → ∞, Theorem 3.2 and the calculations in (5.31)–(5.33) imply that

(5.43) $$1/\lambda_{j}^{\tau }=O\left(e^{\sigma_{j}\tau }\right)\text{ and }\left|\langle q_{i},T_{\tau }q_{l}\rangle \right|\leq \text{min}\left\{e^{-\sigma_{i}\tau },e^{-\sigma_{l}\tau }\right\}.$$

Setting *ϵ* = min {*σ*_*k*+2_ − *σ*_*k*_, *σ*_*k*+1_ – *σ*_*k*−1_} and sending *τ* → ∞, we find that

$$\begin{array}{lll} \left(5.44\right) & d_{\text{F}}\left(\underset{1\leq i\leq k}{\text{span}}\gamma_{i}^{\tau },\underset{1\leq i\leq k}{\text{span}}q_{i}\right) & =\left|\langle q_{k+1},\gamma_{k}\rangle \right|+O\left(e^{-\epsilon \tau }\right)\\ \left(5.45\right) & & =\left|\frac{1}{\lambda_{k}^{\tau }}\sum_{l=1}^{n}\langle q_{k+1},T_{\tau }q_{l}\rangle \langle q_{l},\gamma_{k}^{\tau }\rangle \right|+O\left(e^{-\epsilon \tau }\right). \end{array}$$

Lastly, using the facts that $\gamma_{k}^{\tau }\to q_{k}$ and $\lambda_{k+1}^{\tau }e^{\sigma_{k+1}\tau }\to {\langle \eta_{k+1},q_{k+1}\rangle }^{2}$, we obtain

$$\begin{array}{lll} \left(5.46\right) & d_{\text{F}}\left(\underset{1\leq i\leq k}{\text{span}}\gamma_{i}^{\tau },\underset{1\leq i\leq k}{\text{span}}q_{i}\right)\frac{\lambda_{k}^{\tau }}{\lambda_{k+1}^{\tau }} & =\frac{\langle q_{k+1},T_{\tau }q_{k}\rangle }{\lambda_{k+1}^{\tau }}(1+o(1))\\ \left(5.47\right) & & =\frac{e^{-\sigma_{k+1}\tau }\langle \eta_{k+1},q_{k+1}\rangle \langle \eta_{k+1},q_{k}\rangle }{e^{-\sigma_{k+1}\tau }{\langle \eta_{k+1},q_{k+1}\rangle }^{2}}(1+o(1))\\ \left(5.48\right) & & =\frac{\langle \eta_{k+1},q_{k}\rangle }{\langle \eta_{k+1},q_{k+1}\rangle }(1+o(1)). \end{array}$$

■

### 5.5. Formulas for the estimation error.

In this section, we verify the formulas for the estimation error that are given in Theorem 3.6.

*Proof of Theorem* 3.6. First, define matrices

(5.49) $$\Lambda (\tau )=\text{diag}\left\{\left(\lambda_{1}^{\tau }\;\cdots \;\lambda_{n}^{\tau }\right)\right\},\;V(\tau )=\left(v_{1}(\tau )\;\cdots \;v_{n}(\tau )\right).$$

Due to the normalization $\delta_{ij}=\langle \gamma_{i}^{\tau },\gamma_{j}^{\tau }\rangle$, we must have

(5.50) $$V{\left(\tau \right)}^{T}C(0)V(\tau )=I,\;V{\left(\tau \right)}^{T}C(\tau )V(\tau )=\Lambda (\tau ).$$

Therefore, idealized VAC eigenfunctions $\gamma_{i}^{\tau }$ and eigenvalues $\lambda_{i}^{\tau }$ are the eigenfunctions and eigenvalues of the multiplication operator

(5.51) $$\sum_{i,j=1}^{n}\gamma_{i}^{\tau }\Lambda_{ij}(\tau )\langle \gamma_{j}^{\tau },\cdot \rangle .$$

In contrast, VAC eigenfunctions ${\hat{\gamma }}_{i}^{\tau }$ and eigenvalues ${\hat{\lambda }}_{i}^{\tau }$ are eigenfunctions and eigenvalues of

(5.52) $$\sum_{i,j=1}^{n}\gamma_{i}^{\tau }{\left(V{\left(\tau \right)}^{-1}\hat{C}{\left(0\right)}^{-1}\hat{C}(\tau )V(\tau )\right)}_{ij}\langle \gamma_{j}^{\tau },\cdot \rangle .$$

As $\hat{C}(0)\to C(0)$ and $\hat{C}(\tau )\to C(\tau )$, we can calculate

$$\begin{array}{ll} \left(5.53\right) & C(0)\hat{C}{\left(0\right)}^{-1}\hat{C}(\tau )\\ \left(5.54\right) & ={\left[I+\left[\hat{C}(0)C{\left(0\right)}^{-1}-I\right]\right]}^{-1}\hat{C}(\tau )\\ \left(5.55\right) & =\hat{C}(\tau )-\left[\hat{C}(0)C{\left(0\right)}^{-1}-I\right]C(\tau )+O\left(\|\hat{C}(0)-C(0){\|}_{\text{F}}^{2}+\|\hat{C}(\tau )-C(\tau ){\|}_{\text{F}}^{2}\right)\\ \left(5.56\right) & =V{\left(\tau \right)}^{-T}[\Lambda (\tau )+\hat{L}(\tau )]V{\left(\tau \right)}^{-1}+O\left(\|\hat{C}(0)-C(0){\|}_{\text{F}}^{2}+\|\hat{C}(\tau )-C(\tau ){\|}_{\text{F}}^{2}\right), \end{array}$$

where we have made repeated use of the identities (5.50). Multiplying on the left by *V* (*τ*)^*T*^ and on the right by *V* (*τ*), we find that VAC eigenspaces are unitarily equivalent to the eigenspaces of the matrix operator

(5.57) $$V{\left(\tau \right)}^{-1}\hat{C}{\left(0\right)}^{-1}\hat{C}(\tau )V(\tau )=\Lambda (\tau )+\hat{L}(\tau )+O\left(\|\hat{C}(0)-C(0){\|}_{\text{F}}^{2}+\|\hat{C}(\tau )-C(\tau ){\|}_{\text{F}}^{2}\right),$$

and the two operators share the same eigenvalues. Theorem 3.6 then follows by applying first-order perturbation bounds [15] for eigenvalues and invariant subspaces of a diagonal matrix Λ (*τ*) that is perturbed by a matrix $\hat{L}(\tau )$. ■

### 5.6. Distributional formulas for the estimation error.

In this section, we verify the distributional formulas for the estimation error that are given in Theorem 3.7.

*Proof of Theorem* 3.7. We fix the lag time *t* ≥ 0 and the indices 1 ≤ *i*, *j* ≤ *n*, but allow the total trajectory length *T* to vary. Then, we write

(5.58) $${\hat{C}}_{ij}(t)=\frac{\Delta }{T-t}\sum_{s=0}^{\frac{T-t}{\Delta }-1}\phi_{ij}\left(X_{s\Delta }\right),\;\phi_{ij}(x,y)=\frac{\phi_{i}(x)\phi_{j}(y)+\phi_{i}(y)\phi_{j}(x)}{2}.$$

As *T* →∞, we will proceed to show that $\sqrt{T}\left({\hat{C}}_{ij}(t)-C_{ij}(t)\right)$ converges to an asymptotic normal distribution.

By assumption, *X*_*t*_ is started from the stationary distribution *X*_0_ ~ *μ*, so the random variables ${\left(\phi_{ij}\left(X_{s\Delta },X_{s\Delta +\tau }\right)\right)}_{s=0,1,\ldots }$ are strictly stationary [24, pp. 230–231] with mean *C*_*ij*_(*t*). Moreover, the conditional expectations E [*ϕ*_*ij*_ (*X*_*s*Δ_, *X*_*s*Δ+*τ*_) | *X*_0_ = *x*] satisfy

(5.59) $$\left\|\text{E}\left[\phi_{ij}\left(X_{s\Delta },X_{s\Delta +\tau }\right)|X_{0}=x\right]-C_{ij}(t)\right\|\leq Ce^{-\sigma_{2}s\Delta },\;s\geq 0,$$

for a constant *C* < ∞ that is independent of *s*. Condition (5.59) is an asymptotic negligibility condition that guarantees the validity of the central limit theorem for *ϕ*_*ij*_ *(X*_*s*Δ_, *X*_*s*Δ+*τ*_)*·* Using the central limit theorem in [10, Chap. 5], we prove that

(5.60) $$\sqrt{T}\left({\hat{C}}_{ij}(t)-C_{ij}(t)\right)\overset{D}{\to }N\left(0,\Delta \sum_{s=-\infty }^{\infty }{\text{Cov}}_{\mu }\left[\phi_{ij}^{\tau }\left(X_{0},X_{\tau }\right),\phi_{ij}^{\tau }\left(X_{s\Delta },X_{s\Delta +\tau }\right)\right]\right).$$

For simplicity, we have considered the asymptotic distribution of $\sqrt{T}\left({\hat{C}}_{ij}(t)-C_{ij}(t)\right)$. However, by the same approach we can prove the asymptotic normality of any linear combination of random variables $\sqrt{T}\left({\hat{C}}_{ij}(t)-C_{ij}(t)\right)$ involving different values of *i*, *j*, and *t*. By the Cramér–Wold theorem [14], therefore, the array

(5.61) $$\left[\sqrt{T}(\hat{C}(0)-C(0)),\sqrt{T}(\hat{C}(\tau )-C(\tau ))\right]$$

converges to a mean-zero multivariate normal distribution.

To complete the proof of Theorem 3.7, we then apply the “delta method” [52, p. 26] using the formulas (3.19) and (3.20). Since the $\sqrt{T}\|\hat{C}(0)-C(0){\|}_{\text{F}}^{2}$ and $\sqrt{T}\|\hat{C}(\tau )-C(\tau ){\|}_{\text{F}}^{2}$ terms are $O_{p}\left(\frac{1}{\sqrt{T}}\right)$ as *T* → ∞, these terms are asymptotically negligible. The matrix

(5.62) $$\hat{L}(\tau )=V{\left(\tau \right)}^{T}(\hat{C}(\tau )-\hat{C}(0))V(\tau )-V{\left(\tau \right)}^{T}(\hat{C}(0)-C(0))V(\tau )\Lambda (\tau )$$

is a linear combination of matrices $\hat{C}(\tau )-C(\tau )$ and $\hat{C}(0)-C(0)$, with each matrix entry ${\hat{L}}_{ij}(\tau )$ satisfying

(5.63) $$\sqrt{T}{\hat{L}}_{ij}(\tau )\overset{D}{\to }N\left(0,\Delta \sum_{s=-\infty }^{\infty }{\text{Cov}}_{\mu }\left[F_{ij}^{\tau }\left(X_{0},X_{\tau }\right),F_{ij}^{\tau }\left(X_{s\Delta },X_{s\Delta +\tau }\right)\right]\right).$$

■

Therefore, the formulas (3.19) and (3.20) guarantee the results in Theorem 3.7.

## 6. Conclusions.

In this paper, we have identified and bounded the major error sources of “the variational approach to conformational dynamics” (VAC) [28, 5, 27, 13]. VAC is frequently applied in biomolecular simulation studies to estimate the largest eigenvalues $e^{-\sigma_{1}\tau }\geq e^{-\sigma_{2}\tau }\geq \cdots \geq e^{-\sigma_{k}\tau }$ for the Markov transition operator *T*_*τ*_, along with the corresponding eigenfunctions *η*_1_, *η*_2_, … , *η*_*k*._

We have proved that VAC accurately identifies subspaces of eigenfunctions span_*j*≤*i*≤*k*_ *η*_*i*_ when three conditions are satisfied:

1. The values {*σ*_*j*_, … , *σ*_*k*_} are separated from all other *σ*_*i*_ values by a spectral gap.
2. The library of basis functions (*ϕ*_*i*_)_1≤*i*≤*n*_ becomes very rich so that linear combinations of basis functions can fully represent *η*_1_, … , *η*_*k*_.
3. The data set becomes very large so that expectations *C*_*ij*_(0) = *E*_*μ*_ [*ϕ*_*i*_ (*X*_0_) *ϕ*_*j*_ (*X*_0_) and *C*_*ij*_ (*τ*) = E_*μ*_ [*ϕ*_*i*_ (*X*_0_) *ϕ*_*j*_ (*X*_*τ*_)] are evaluated with vanishing error.

VAC converges for any value of the lag time parameter *τ* > 0, yet the choice of lag time can dramatically alter the speed of convergence. Hence, our main contribution is to prove error bounds that explicitly show how error depends on the lag time. These bounds provide a full theoretical justification for why limitations in the basis set contribute to the error at short lag times and limitations in the data set contribute to the error at long lag times.

Our numerical analysis approach is flexible, and it could be extended to algorithms besides VAC that estimate dynamical quantities of interest using trajectory data. A broadly useful approach involves decomposing the total error into approximation error and estimation error. Another useful approach involves identifying asymptotic formulas for the estimation error. In future research, it is our goal to rigorously analyze the approximation and estimation error for other powerful algorithms used in biochemical simulation (e.g., [49]).

Lastly, while the main purpose of our work is to deepen theoretical understanding, we also provide diagnostic tools to assess VAC’s estimation error and tune VAC’s parameters to reduce this error source. We present the *VAC condition number* as a tool for identifying subspaces of VAC eigenfunctions that are prone to experiencing high estimation error. We also present the *mean squared estimation error* as a tool for calculating the estimation error at different lag times. Motivated by the present study, we have also developed an approach for reducing the lag time sensitivity and increasing VAC’s robustness by integrating over a window of lag times [20]. These tools have direct relevance to the researchers using VAC, pointing the way toward a more streamlined lag time selection process and a more critical assessment of VAC’s error for the future.

## Supplementary Material

Supplementary Methods and Further Details
