# Supplementary material for: Error Bounds for Dynamical Spectral Estimation
Source: SIAM J Math Data Sci. Author manuscript; Available in PMC 2021 Aug 4. (PMC8336423; doi:10.1137/20m1335984)
Supplement: Supplementary Methods and Further Details [file NIHMS1680331-supplement-Supplementary_Methods_and_Further_Details.pdf]

SUPPLEMENTARY MATERIALS: Error Bounds for Dynamical Spectral Estimation*

Robert J. Webber[†], Erik Thiede[‡], Douglas Dow[§], Aaron Dinner[‡], and Jonathan Weare[†]

SM1. Figures for the Ornstein-Uhlenbeck process.

Here we provide additional information about how Figure 2 - Figure 5 were generated. These figures show VAC applied to the Ornstein-Uhlenbeck process

$$dX = -X\,dt + \sqrt{2}\,dW\,. \tag{SM1.1}$$

started from the stationary $\mathcal{N}(0,1)$ distribution. The eigenfunctions of the transition operator T_t are the Hermite polynomials

$$1, x, \frac{x^2-1}{\sqrt{2}}, \frac{x^3-3x}{\sqrt{6}}, \ldots \tag{SM1.2}$$

with eigenvalues $1, e^{-t}, e^{-2t}, e^{-3t}, \ldots$.

The conditional distribution for the OU process is determined by

$$\text{Law}\left(X_t \middle| X_0 = x\right) = \mathcal{N}\left(xe^{-t}, 1-e^{-2t}\right). \tag{SM1.3}$$

Therefore, we can simulate the OU process in discrete time using the exact evolution equations

$$X_{t+\Delta} = e^{-\Delta} X_t + \xi_t, \quad \xi_t \sim \mathcal{N}\left(0, 1-e^{-2\Delta t}\right). \tag{SM1.4}$$

When we apply VAC to the OU process, we use a basis of n indicator functions on disjoint intervals, namely,

$$\left\{\mathbb{1}_{(-\infty,q_1)}, \mathbb{1}_{[q_1,q_2)}, \mathbb{1}_{[q_2,q_3)}, \ldots, \mathbb{1}_{[q_{n-1},\infty)}\right\}. \tag{SM1.5}$$

The boundary points

$$q_0 = -\infty < q_1 < q_2 < \cdots < q_{n-1} < q_n = \infty \tag{SM1.6}$$

are selected as follows:

---

*Supplementary material for SIMODS MS#M133598.
http://www.siam.org/journals/10.1137/20M1335984

[†]Courant Institute of Mathematical Sciences, New York University, New York, NY 10012 (rw2515@nyu.edu, weare@nyu.edu).
[‡]Department of Chemistry, University of Chicago, Chicago, IL 60637 (thiede@uchicago.edu, dinner@uchicago.edu).
[§]Department of Mathematics, University of Chicago, Chicagoo, IL 60637 (ddow11@uchicago.edu).

1. First, we set $q_i = \Phi^{-1}(i/n)$ where

$$\Phi(x) = \int_{-\infty}^{x} \frac{e^{-y^2/2}}{\sqrt{2\pi}}\, dy \tag{SM1.7}$$

is the cumulative distribution function for a standard normal random variable, and Φ^{-1} is the inverse cumulative distribution function, also called the quantile function.
2. Next, we set $q_i \leftarrow q_i + \epsilon$, where ϵ is an offset parameter that is always $\epsilon = 0.1$ in our figures. The offset parameter helps make our examples realistic, since it would typically be impossible in VAC applications to identify quantiles of the equilibrium distribution exactly.

Although many quantities involving the Ornstein-Uhlenbeck process can be calculated analytically, we used numerical quadrature to evaluate the integrals

$$\langle \eta_i, \phi_j \rangle = \int_{q_{j-1}}^{q_j} \eta_i(x) \frac{e^{-x^2/2}}{\sqrt{2\pi}}\, dx \tag{SM1.8}$$

and

$$\langle \phi_i, T_\tau \phi_j \rangle = \int_{q_{j-1}}^{q_j} \left[\Phi\left(\frac{q_i - x e^{-\tau}}{\sqrt{1 - e^{-2\tau}}} \right) - \Phi\left(\frac{q_{i-1} - x e^{-\tau}}{\sqrt{1 - e^{-2\tau}}} \right) \right] \frac{e^{-x^2/2}}{\sqrt{2\pi}}\, dx\,. \tag{SM1.9}$$

SM2. Figures for the double well process. Here we provide additional information about how Figure 6 and Figure 7 were generated. These figures show VAC applied to the process

$$dX = -\frac{1}{2}\sigma\sigma^T \nabla U(X)\, dt + \sigma\, dW, \tag{SM2.1}$$

where the potential U and the diffusion matrix σ are given by:

$$U(x_1, x_2) = 4x_1^4 - 8x_1^2 + x_1 + 0.5x_2^2, \qquad \sigma = \begin{pmatrix} 2 & 0 \\ -1 & \sqrt{3} \end{pmatrix}. \tag{SM2.2}$$

X_t is a double well process that spends long time periods in potential wells near $(-1, 0)$ and $(1, 0)$ with rare transitions between wells. We simulate X_t using the BAOAB-limit integrator presented in Leimkuhler and Matthews [SM3] with the timestep $\Delta = 10^{-4}$. We discard the first $t = 10$ time units of each trajectory to reduce equilibration bias.

To calculate reference values for the true eigenfunctions η_i and the idealized VAC matrices $C(\tau)$, we use the numerical PDE approach from appendix D of reference [SM5]. We first construct a grid from -2 to 2 in x and from -5 to 5 in y with grid spacing of $\epsilon = \left(6 \times 10^{-4}\right)^{-1/2}$. We next construct the transition matrix P for a hopping process on a grid:

$$P(x \pm \epsilon, y) = \frac{1}{6\left(1 + \exp\left[U(x \pm \epsilon, y) - U(x, y)\right]\right)}, \tag{SM2.3}$$

$$P(x, y \pm \epsilon) = \frac{1}{6\left(1 + \exp\left[U(x, y \pm \epsilon) - U(x, y)\right]\right)}, \tag{SM2.4}$$

$$P(x \pm \epsilon, y \pm \epsilon) = \frac{1}{6\left(1 + \exp\left[U(x \pm \epsilon, y \pm \epsilon) - U(x, y)\right]\right)}, \tag{SM2.5}$$

$$P(x \pm \epsilon, y \mp \epsilon) = 0, \tag{SM2.6}$$

$$P(x, y) = 1 - P(x + \epsilon, y) - P(x - \epsilon, y) - P(x, y + \epsilon) - P(x, y - \epsilon). \tag{SM2.7}$$

In the $\epsilon \to 0$ limit, the action of $\frac{24}{\epsilon^2}(P - I)$ on smooth functions approximates the action of the infinitesimal generator L for the process X_t.

We calculate the eigenfunctions η_i using eigenfunctions of P. We calculate the idealized VAC matrix $C(\tau)$ using the approximation

$$C_{ij}(\tau) = \left\langle \phi_i, e^{L\tau} \phi_j \right\rangle \tag{SM2.8}$$

$$\approx \left\langle \phi_i, \left(I + \frac{\epsilon^2}{24} L\right)^{24\tau/\epsilon^2} \phi_j \right\rangle \tag{SM2.9}$$

$$\approx \vec{\phi}_i^T D_\mu P^{24\tau/\epsilon^2} \vec{\phi}_j, \tag{SM2.10}$$

where $\vec{\phi}_i$ is the vector of ϕ_i values evaluated at each gridpoint and D_μ is a diagonal matrix with the stationary measure at each gridpoint along the diagonal.

SM3. Mean squared estimation error. In Algorithm SM3.1, we describe the procedure for calculating the mean squared estimation error using data. The procedure is similar to the one used to calculate error bars in Markov chain Monte Carlo. Therefore, we take advantage of existing software for Markov chain Monte Carlo sampling [?] in our implementation.

SM4. Rayleigh-Ritz approximation bounds. In this section, we re-derive the classic approximation bounds for the Rayleigh-Ritz method first presented in [SM1] and [SM2, pg. 992]. Our first step is to verify the inequality

$$1 - d_2^2\left(\operatorname*{span}_{1 \leq i \leq k} \eta_i, \Phi\right) \leq \frac{\lambda_k^\tau}{e^{-\sigma_k \tau}} \leq 1 \tag{SM4.1}$$

that appears in the statement of Theorem 3.2.

Proof of equation (3.5). As in the proof of Theorem 3.4, the upper bound

$$\lambda_k^\tau = \max_{\dim(\mathrm{H}) = k, \mathrm{H} \subseteq \Phi} \min_{\eta \in \mathrm{H}} \frac{\langle \eta, T_\tau \eta \rangle}{\langle \eta, \eta \rangle} \leq e^{-\sigma_k \tau} \tag{SM4.2}$$

follows directly from the min-max principle.

The lower bound on λ_k^τ follows trivially if $d_2\left(\operatorname{span}_{1 \leq i \leq k} \eta_i, \Phi\right) = 1$. If $d_2\left(\operatorname{span}_{1 \leq i \leq k}, \Phi\right) < 1$, then we define subspaces $\mathrm{H}_{1:k} = \operatorname{span}_{1 \leq i \leq k} \eta_i$ and $Q_{1:k} = P_\Phi \operatorname{span}_{1 \leq i \leq k} \eta_i$. For any $q \in Q_{1:k}$ with $\|q\| = 1$, we calculate

$$e^{\sigma_k \tau} \leq \frac{\langle T_\tau P_{\mathrm{H}_{1:k}} q, P_{\mathrm{H}_{1:k}} q \rangle}{\langle P_{\mathrm{H}_{1:k}} q, P_{\mathrm{H}_{1:k}} q \rangle} \tag{SM4.3}$$

$$= \frac{\langle T_\tau P_{\mathrm{H}_{1:k}} q, P_{\mathrm{H}_{1:k}} q \rangle}{1 - \left\| P_{\mathrm{H}_{1:k}^\perp} q \right\|^2} \tag{SM4.4}$$

$$\leq \frac{\langle P_{\mathrm{H}_{1:k}} T_\tau P_{\mathrm{H}_{1:k}} q, q \rangle + \left\langle P_{\mathrm{H}_{1:k}^\perp} T_\tau P_{\mathrm{H}_{1:k}^\perp} q, q \right\rangle}{1 - \left\| P_{\mathrm{H}_{1:k}^\perp} P_{Q_{1:k}} \right\|^2} \tag{SM4.5}$$

$$= \frac{\langle T_\tau q, q \rangle}{1 - d_2^2\left(\mathrm{H}_{1:k}, \Phi\right)}. \tag{SM4.6}$$

Algorithm SM3.1 Asymptotic estimation error.

1. For $1 \leq i, j \leq n$, perform the following calculations.
   (a) Form the time series $\hat{F}_{ij}^{\tau}\left(X_{s\Delta}, X_{s\Delta+\tau}\right)$ for $s = 0, 1, \ldots, \frac{T-\tau}{\Delta} - 1$, where the function $\hat{F}_{ij}^{\tau}(x, y)$ is given by

$$\frac{\hat{\gamma}_i^{\tau}(x)\,\hat{\gamma}_j^{\tau}(y) + \hat{\gamma}_i^{\tau}(y)\,\hat{\gamma}_j^{\tau}(x)}{2} - \hat{\lambda}_j^{\tau}\frac{\hat{\gamma}_i^{\tau}(x)\,\hat{\gamma}_j^{\tau}(x) + \hat{\gamma}_i^{\tau}(y)\,\hat{\gamma}_j^{\tau}(y)}{2}. \tag{SM3.1}$$

   (b) For $s = 0, 1, \ldots, \frac{T-\tau}{\Delta} - 1$, calculate the autocovariance terms $\hat{R}_{ij}(s\Delta)$ given by

$$\frac{1}{\frac{T-\tau}{\Delta} - s}\sum_{r=0}^{\frac{T-\tau}{\Delta}-s-1} \hat{F}_{ij}^{\tau}\left(X_{r\Delta}, X_{r\Delta+\tau}\right)\hat{F}_{ij}^{\tau}\left(X_{(r+s)\Delta}, X_{(r+s)\Delta+\tau}\right). \tag{SM3.2}$$

   (c) Use the approach in [SM4, pg.143-145] to determine a truncation threshold K such that $\hat{R}_{ij}(s\Delta) \approx 0$ for $s > K$, and set

$$\hat{V}_{ij}(\tau)^2 = \frac{\Delta}{T}\left(1 + 2\sum_{s=1}^{K}\hat{R}_{ij}(s\Delta)\right). \tag{SM3.3}$$

2. Estimate the mean squared estimation error using

$$\mathrm{E}\left|\hat{\lambda}_i^{\tau} - \lambda_i^{\tau}\right|^2 \approx \hat{V}_{ii}(\tau)^2, \tag{SM3.4}$$

$$\mathrm{E}\left|d_{\mathrm{F}}\left(\operatorname*{span}_{j\leq i\leq k}\hat{\gamma}_i^{\tau}, \operatorname*{span}_{j\leq i\leq k}\gamma_i^{\tau}\right)\right|^2 \approx \sum_{\substack{l<j \\ \text{or } l>k}}\sum_{m=j}^{k}\frac{\hat{V}_{lm}(\tau)^2}{\left|\hat{\lambda}_l^{\tau} - \hat{\lambda}_m^{\tau}\right|^2}. \tag{SM3.5}$$

We conclude that

$$\left(1 - d_2^2\left(\mathrm{H}_{1:k}, \Phi\right)\right)e^{\sigma_k\tau} \leq \frac{\langle q, T_\tau q\rangle}{\langle q, q\rangle}, \quad q \in Q_{1:k}. \tag{SM4.7}$$

The lower bound then follows by applying the min-max principle. ∎

It remains to verify the inequality

$$1 \leq \frac{d_{\mathrm{F}}^2\left(\operatorname*{span}_{1\leq i\leq k}\gamma_i^{\tau}, \operatorname*{span}_{1\leq i\leq k}\eta_i\right)}{d_{\mathrm{F}}^2\left(\operatorname*{span}_{1\leq i\leq k}\eta_i, \Phi\right)} \leq 1 + \frac{\left\|P_{\Phi^\perp}T_\tau P_\Phi\right\|_2^2}{\left|e^{-\sigma_k\tau} - \lambda_{k+1}^{\tau}\right|^2} \tag{3.6}$$

that appears in the statement of Theorem 3.2.

Proof of equation (3.6). We define subspaces $\mathrm{H}_{j:k} = \mathrm{span}_{j\leq i\leq k}\, \eta_i$ and $\Gamma^\tau_{j:k} = \mathrm{span}_{j\leq i\leq k}\, \gamma^\tau_i$ for all $1 \leq j < k \leq n$. Then, it follows

$$1 \leq \frac{d_{\mathrm{F}}^2\left(\operatorname*{span}_{1\leq i\leq k} \gamma_i^\tau, \operatorname*{span}_{1\leq i\leq k} \eta_i\right)}{d_{\mathrm{F}}^2\left(\operatorname*{span}_{1\leq i\leq k} \eta_i, \Phi\right)} = \frac{\left\|P_{\mathrm{H}_{1:k}} P_{(\Gamma^\tau_{1:k})^\perp}\right\|_{\mathrm{F}}^2}{\|P_{\mathrm{H}_{1:k}} P_{\Phi^\perp}\|_{\mathrm{F}}^2} \leq 1 + \frac{\left\|P_{\mathrm{H}_{1:k}} P_{\Gamma^\tau_{k+1:n}}\right\|_{\mathrm{F}}^2}{\|P_{\mathrm{H}_{1:k}} P_{\Phi^\perp}\|_{\mathrm{F}}^2}. \tag{SM4.8}$$

To bound the distance between $\mathrm{H}_{1:k}$ and the idealized VAC subspace $\Gamma^\tau_{k+1:n}$, we apply the Davis-Kahan lemma as in the proof of Theorem 3.4. The spectrum of $P_{\mathrm{H}_{1:k}}\, T_\tau|_{\mathrm{H}_{1:k}}$ lies in $[e^{-\sigma_k\tau}, \infty)$, while the spectrum of $P_{\Gamma^\tau_{k+1:n}}\, T_\tau|_{\Gamma^\tau_{k+1:n}}$ lies in $\left(-\infty, \lambda^\tau_{k+1}\right]$. Therefore, the spectral gap is at least $e^{-\sigma_k\tau} - \lambda^\tau_{k+1}$. It follows that

$$\left(e^{-\sigma_k\tau} - \lambda^\tau_{k+1}\right) \left\|P_{\mathrm{H}_{1:k}} P_{\Gamma^\tau_{k+1:n}}\right\|_{\mathrm{F}} \leq \left\|P_{\mathrm{H}_{1:k}} P_{\Gamma^\tau_{k+1:n}} T_\tau P_{\Gamma^\tau_{k+1:n}} - P_{\mathrm{H}_{1:k}} T_\tau P_{\mathrm{H}_{1:k}} P_{\Gamma^\tau_{k+1:n}}\right\|_{\mathrm{F}} \tag{SM4.9}$$

$$= \left\|P_{\mathrm{H}_{1:k}} P_{\Phi} T_\tau P_{\Gamma^\tau_{k+1:n}} - P_{\mathrm{H}_{1:k}} T_\tau P_{\Gamma^\tau_{k+1:n}}\right\|_{\mathrm{F}} \tag{SM4.10}$$

$$= \left\|P_{\mathrm{H}_{1:k}} P_{\Phi^\perp} T_\tau P_{\Gamma^\tau_{k+1:n}}\right\|_{\mathrm{F}} \tag{SM4.11}$$

$$\leq \|P_{\mathrm{H}_{1:k}} P_{\Phi^\perp}\|_{\mathrm{F}} \, \|P_{\Phi^\perp} T_\tau P_\Phi\|_2\,. \tag{SM4.12}$$

where we have used the fact that $\Gamma^\tau_{k+1:n}$ is an invariant subspace of $P_\Phi T_\tau P_\Phi$ and $\mathrm{H}_{1:k}$ is an invariant subspace of τ. ∎

SM5. Sharper bounds on the lag-time-independent error. Here we prove an elegant bound on the lag-time-independent error.

Proposition SM5.1. *The lag-time-independent error satisfies*

$$1 \leq \frac{d_{\mathrm{F}}^2\left(\operatorname*{span}_{j\leq i\leq k} q_i, \operatorname*{span}_{j\leq i\leq k} \eta_i\right)}{d_{\mathrm{F}}^2\left(\operatorname*{span}_{j\leq i\leq k} \eta_i, \Phi\right)} \leq \frac{1}{1 - d_2^2\left(\operatorname*{span}_{1\leq i\leq j-1} \eta_i, \Phi\right)}. \tag{SM5.1}$$

Proof. To verify the upper bound, it is enough to prove

$$\left\|P_{Q^\perp_{j:k}} \eta\right\|^2 \leq \frac{\|P_{\Phi^\perp}\eta\|^2}{1 - \left\|P_{\Phi^\perp} P_{\mathrm{H}_{1:j-1}}\right\|_2^2}, \quad \eta \in \mathrm{H}_{j:k}\,. \tag{SM5.2}$$

Moreover, observing that

$$\left\|P_{Q^\perp_{j:k}} \eta\right\|^2 = \left\|P_{Q_{1:j-1}} \eta\right\|^2 + \|P_{\Phi^\perp}\eta\|^2\,, \tag{SM5.3}$$

it is enough to prove

$$\left\|P_{Q_{1:j-1}} \eta\right\|^2 = \left\|P_{Q^\perp_{j:k}} \eta\right\|^2 \leq \frac{\|P_{\Phi^\perp}\eta\|^2 \left\|P_{\Phi^\perp} P_{\mathrm{H}_{1:j-1}}\right\|_2^2}{1 - \left\|P_{\Phi^\perp} P_{\mathrm{H}_{1:j-1}}\right\|_2^2}, \quad \eta \in \mathrm{H}_{j:k}\,. \tag{SM5.4}$$

If $P_{Q_{1:j-1}}\eta = 0$, then equation (SM5.4) follows trivially. Therefore, we consider $\eta \in \mathrm{H}_{j:k}$ such that $P_{Q_{1:j-1}}\eta \neq 0$. Then, there is a function $\eta' \in \mathrm{H}_{1:j-1}$ with

$$P_{\Phi}\eta' = \frac{P_{Q_{1:j-1}}\eta}{\left\|P_{Q_{1:j-1}}\eta\right\|}. \tag{SM5.5}$$

We can bound the norm of η' by observing

$$\left\|\eta'\right\|^2 = \left\|P_{\Phi}\eta'\right\|^2 + \left\|P_{\Phi^\perp}\eta'\right\|^2 \leq 1 + \left\|P_{\Phi^\perp}P_{\mathrm{H}_{1:j-1}}\right\|_2^2 \left\|\eta'\right\|^2. \tag{SM5.6}$$

This gives the norm bound

$$\left\|\eta'\right\|^2 \leq \frac{1}{1 - \left\|P_{\Phi^\perp}P_{\mathrm{H}_{1:j-1}}\right\|_2^2 \left\|\eta'\right\|^2}. \tag{SM5.7}$$

Using the norm bound and the orthogonality of $\eta \in \mathrm{H}_{j:k}$ and $\eta' \in \mathrm{H}_{1:j-1}$, we conclude

$$\begin{aligned}
\left\|P_{Q_{1:j-1}}\eta\right\|^2 &= \left\|\left\langle P_{Q_{1:j-1}}\eta, P_{\Phi}\eta'\right\rangle P_{\Phi}\eta'\right\|^2 && \text{(SM5.8)}\\
&= \left\langle P_{Q_{1:j-1}}\eta, P_{\Phi}\eta'\right\rangle^2 && \text{(SM5.9)}\\
&= \left\langle P_{\Phi}\eta, P_{\Phi}\eta'\right\rangle^2 && \text{(SM5.10)}\\
&= \left\langle P_{\Phi^\perp}\eta, P_{\Phi^\perp}\eta'\right\rangle^2 && \text{(SM5.11)}\\
&\leq \left\|P_{\Phi^\perp}\eta\right\|^2 \left\|P_{\Phi^\perp}\eta'\right\|^2 && \text{(SM5.12)}\\
&\leq \frac{\left\|P_{\Phi^\perp}\eta\right\|^2 \left\|P_{\Phi^\perp}P_{\mathrm{H}_{1:j-1}}\right\|_2^2}{1 - \left\|P_{\Phi^\perp}P_{\mathrm{H}_{1:j-1}}\right\|_2^2}. && \text{(SM5.13)}
\end{aligned}$$

■

REFERENCES

[1] A. Knyazev, *Sharp a priori error estimates of the Rayleigh-Ritz method without assumptions of fixed sign or compactness*, Mathematical Notes, 38 (1985), pp. 998–1002.

[2] A. Knyazev, *New estimates for Ritz vectors*, Mathematics of Computation, 66 (1997), pp. 985–995.

[3] B. Leimkuhler and C. Matthews, *Rational construction of stochastic numerical methods for molecular sampling*, Applied Mathematics Research eXpress, 2013 (2013), pp. 34–56.

[4] A. Sokal, *Monte Carlo Methods in Statistical Mechanics: Foundations and New Algorithms*, in Functional Integration, Springer, 1997, pp. 131–192.

[5] E. H. Thiede, D. Giannakis, A. R. Dinner, and J. Weare, *Galerkin approximation of dynamical quantities using trajectory data*, The Journal of Chemical Physics, 150 (2019), p. 244111.
